# Tracking Oxidized Volatile Methyl Siloxanes in New York City in Summertime

**DOI:** 10.1021/acsestair.6c00134

**Published:** 2026-09-03

**Authors:** Jeewani N. Meepage, Josie K. Welker, Christopher E. Brunet, Charles O. Stanier, Elizabeth A. Stone

**Affiliations:** † Department of Chemistry, University of Iowa, Iowa City, Iowa 52242, United States; ‡ Department of Civil and Environmental Engineering, IIHR-Hydroscience & Engineering, University of Iowa, Iowa City, Iowa 52242, United States; § Department of Chemical and Biochemical Engineering, University of Iowa, Iowa City, Iowa 52242, United States

**Keywords:** secondary organic aerosol, personal care products, urban air quality, gas particle partitioning, sampling artifacts, siloxanol, SOA tracer

## Abstract

Volatile methyl siloxanes (VMS) are high-production anthropogenic chemicals that can undergo atmospheric oxidation to form oxidized VMS (oVMS) in the atmosphere and contribute to secondary organic aerosol (SOA). Herein, we develop an SOA tracer-based approach to estimate oVMS contributions to ambient fine particulate matter (PM_2.5_) organic carbon (OC) in New York City (NYC). oVMS including a series of siloxanols with intact D_4_, D_5_, and D_6_ rings were identified and semiquantified in field and laboratory chamber aerosol samples by liquid chromatography–tandem mass spectrometry. oVMS were persistent in PM_2.5_ in NYC during summertime, with oVMS from D_4_ and D_6_ showing more atmospheric aging than D_5_. Statistically significant correlations among these oVMS suggest common atmospheric formation pathways. Daytime concentrations of oVMS were significantly higher than nighttime levels consistent with a photochemical source. In PM_2.5_, the most prominent oVMS signal was the disiloxanol which partitioned primarily to the particle phase (averaging 77%). Using the tracer-based approach, D_4,_ D_5_, and D_6_-derived SOA was estimated to contribute 14 ± 5 ng m^–3^ to PM_2.5_ corresponding to ∼0.10% of PM_2.5_ organic carbon, making VMS oxidation a minor source of PM_2.5_ in NYC.

## Graphical abstract

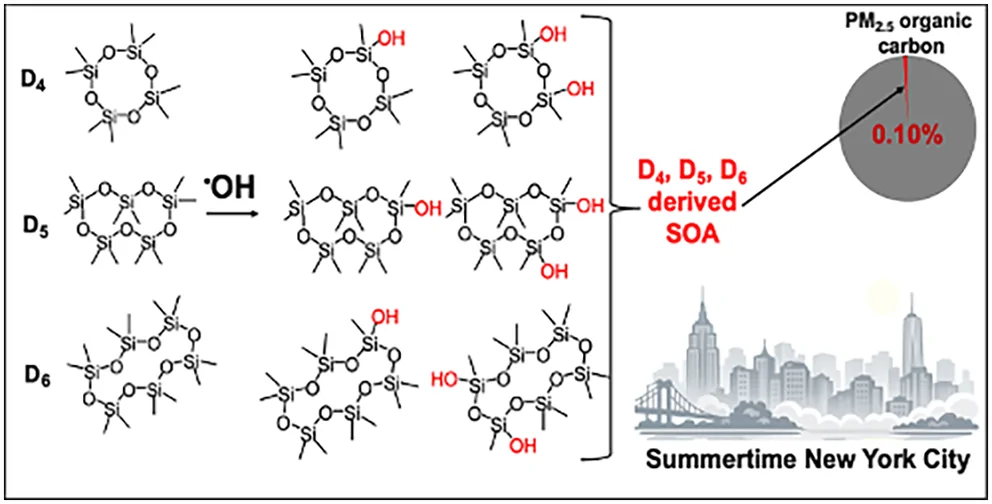

## 1 Introduction

Volatile methyl siloxane (VMS) global production volume exceeds 2 million metric tons per year, with VMS exhibiting unique properties that include low surface tension, high thermal stability, flexibility, and volatility.^1–3^ As a result, VMS are used in a wide range of consumer and industrial products.^4,5^ The most abundant VMS in the atmosphere are octamethylcyclotetrasiloxane (D_4_), decamethylcyclopentasiloxane (D_5_), and dodecamethylcyclohexasiloxane (D_6_).^4,6^ D_5_ and D_6_ are predominantly associated with personal care products, such as antiperspirants, lotions, and hair care products, while D_4_ is primarily an industrial chemical used in the manufacturing of polymers, adhesives, and building materials.^7,8^ Due to their high vapor pressure, approximately 90% of VMS rapidly volatilize into the atmosphere, leading to their accumulation in indoor air and subsequent transport to the outdoor air.^9,10^ The widespread occurrence of VMS has raised concerns regarding their potential for bioaccumulation and adverse impacts on human and environmental health. D_4_ and D_5_ are classified as bioaccumulative or very bioaccumulative compounds, while D_6_ exhibits a lower bioaccumulation potential.^4^ Toxicological studies have linked D_4_ exposure to impaired fertility and liver and lung damage, while D_5_ has been associated with carcinogenic effects, including uterine tumors in laboratory studies.^6,11^ VMS are now ubiquitous in indoor and outdoor environments, raising concerns regarding their atmospheric fate and potential contributions to secondary organic aerosol (SOA).^12^ This study evaluates the composition and extent of VMS-derived SOA in urban New York City (NYC) during summertime, with a focus on its contribution to fine-particle (PM_2.5_) organic carbon (OC).

In the atmosphere, VMS react primarily with hydroxyl radicals (OH).^13,14^ For a 24-h average OH concentration of 1.2 × 10^6^ molecules cm^–3^, lifetimes for D_4_–D_6_ range from approximately 3 to 10 days, enabling extended reaction time and long-range transport.^13–15^ Oxidized VMS (oVMS) can be less volatile and more polar than their precursor gases, and can partition into particles and form SOA.^16–24^ Numerous laboratory studies have examined the chemical composition of oVMS from D_5_,^20–23,25–27^ while fewer have focused on D_4_.^20,28^ In the case of D_5_ oxidation, the principal first-generation product observed in chamber and flow reactor experiments is 1-hydroxynonamethylcyclopentasiloxane (D_4_TOH, D_5_ siloxanol hereafter), formed by substitution of a methyl group by OH.^17,20,21^ In addition, oVMS can include multiple oxygenated functional groups (e.g., siloxanol and silyl methanol), ring-opened oxidized compounds, and higher molecular weight dimers linked through oxygen or methylene bridges.^20–22,29,30^ The partitioning behavior of early generation oxidation products such as D_5_ siloxanol depends on factors including seed aerosol composition, aerosol loading, humidity, and temperature, making the extent of SOA formation dependent on local conditions.^17,30^

Evidence of VMS-derived SOA in ambient air has emerged from recent field studies of oVMS in urban PM_2.5_, although these measurements are semiquantitative due to the lack of authentic oVMS standards.^27,31,32^ At two urban sites in the United States, D_5_ siloxanol was detected at <0.2 ng m^–3^.^31^ Previously, in NYC, D_5_ siloxanol, disiloxanols, and tetrasiloxanols were observed, suggesting that multiple OH oxidation steps can occur in the formation of particle phase oVMS.^27,32^ Sampling artifacts can significantly impact oVMS measurements, through sorption to filter media (i.e., those made of quartz fiber), and should be considered in field measurements.^32^ In Beijing, China, water-soluble Si has been used as a proxy for silicon-containing SOA, indicating that Si-SOA accounted for 0.06 and 0.16% of PM_2.5_, and reached up to 0.26 and 0.92% on certain days in summer and winter, respectively.^33^ These observations, while sparse, indicate that oVMS can contribute to urban PM_2.5_, especially in areas with substantial VMS emissions. Modeling studies have also examined the potential SOA contribution of oVMS. Janechek et al.^19^ applied the Community Multiscale Air Quality model (CMAQ) with D_4_, D_5_, and D_6_ precursors and their oxidation products, estimating that urban concentrations of oxidized D_5_ can reach up to 9 ng m^–3^ in cities like NYC. Pennington et al.^34^ estimated that siloxanes formed very little SOA compared to other components of volatile chemical products, such as alkanes, aromatics, benzyl alcohol, and glycol ethers. Han et al.^24^ used empirical yield models to estimate that oxidation of cyclic VMS could contribute to a global SOA source strength of approximately 0.01 Tg year^–1^, concentrated in urban regions. The relative importance of volatile chemical products to SOA formation is expected to increase as transportation and industrial VOC emissions continue to decline, positioning oVMS as contributors to SOA of growing importance.^12^ Furthermore, oVMS are ideal tracers of volatile chemical product emissions, enabling more precise constraints on their contributions to SOA and PM_2.5_.^8^ Collectively, these studies highlight the potential for oVMS to contribute to urban PM but lack quantitative field measurements.

With a focus on the urban atmosphere of NYC during summertime, the current study assesses VMS (D_4_, D_5_, and D_6_) contributions to PM_2.5_ OC using the SOA tracer approach for the first time and characterizes ambient oVMS trends. Our specific objectives include (1) identification and semiquantitative analysis of VMS-derived oVMS in gas and PM_2.5_ samples; (2) evaluation of gas particle distributions; (3) characterization of the temporal variations in oVMS concentrations in NYC; and (4) integration of field measurements with data from laboratory chamber experiments to estimate D_4_, D_5_, and D_6_ contributions to SOA mass and PM_2.5_ OC. Building upon our prior work on D_5_,^27^ this study extends the analysis to oVMS derived from D_4_ and D_6_ in the ambient atmosphere. Through a month of continuous diurnal measurements of oVMS in NYC during summer 2022, this study presents the first compound-resolved, observation-constrained estimates of VMS-derived SOA in urban air.

## 2 Materials and Methods

### 2.1 Chemicals and Reagents

Because oVMS standards were not commercially available at the time of this study, four commercially available organosilicon compounds were used as surrogate standards: tris­(tert-butoxy)­silanol (99.999%, Sigma-Aldrich), tris­(tert-pentoxy)­silanol (99.99%, Sigma-Aldrich), dimethylphenylsilanol (95%, Sigma-Aldrich), and t-butyldimethylsilanol (99%, Sigma-Aldrich). Among these, all four served as spike recovery surrogates while tris­(tert-butoxy)­silanol was used as the quantification surrogate. D_5_-phenol (98%, Sigma-Aldrich) was used as an internal standard. Acetonitrile was purchased from Fisher Scientific (Optima), and ultrapure water (18.2 MΩ-cm) was generated onsite (Thermo, Barnstead EasyPure-II). Ammonia (20–22% as NH_3_, Fisher Scientific) and ammonium bicarbonate (99.0%, Sigma-Aldrich) were used to prepare the mobile phase buffer. Ammonium sulfate (99.0%, Sigma-Aldrich), D_5_ (97%, Thermo Fisher Scientific), and hydrogen peroxide (30%, Fisher Scientific) were used in the chamber experiments described here.

### 2.2 NYC-METS 2022 Field Measurements

#### 2.2.1 Field Site Description

Ambient samples were collected from the rooftop of the City University of New York’s Advanced Science Research Center in northern Manhattan, New York City, USA (40°48′55.5″N, 73°57′01.5″W) from July 7 to August 3, 2022, during the 2022 NYC-METS (New York City-metropolitan Measurements of Emissions and TransformationS) campaign. Situated in a densely populated urban area, the measurement site is surrounded by residential buildings, community buildings such as universities, schools, and city streets, placing it close to a wide range of urban VMS sources.

#### 2.2.2 Collocated Measurements

The NYC-METS field campaign in 2022 provided access to numerous collocated measurements that were previously described in the literature. Brunet et al.^7^ reported gas-phase measurements of D_4_, D_5_, and D_6_ by gas chromatography with triple quadrupole mass spectrometry. Planetary boundary layer heights were obtained for the study site from the NOAA High-Resolution Rapid Refresh (HRRR) model archives (3 km, 1 h).^35^ Hass-Mitchell et al.^36^ reported on-site measurements of temperature, relative humidity, rain intensity, and rainfall accumulation (Vaisala HMP155 sensor); wind speeds (RM Young 81000 Sonic Anemometer, 1 min resolution); carbon monoxide (CO) by tunable infrared laser direct absorption spectroscopy (TILDAS, Aerodyne); ozone (2B Technologies Model 202); and nitrogen oxides (NO and NO_2_, 2B Technologies Model 405 monitor). All collocated measurements were time-averaged to correspond to the PM sampling periods.

#### 2.2.3 Aerosol and Gas Sample Collection

Ambient PM_2.5_ samples were collected as described in Welker et al.^32^ following a daytime (D: 08:00–20:00 EDT) and nighttime (N: 20:00–08:00 EDT) schedule. Samples were collected using two collocated medium-volume air samplers (cyclone with an aerodynamic diameter of <2.5 μm, URG-3000B, URG Corporation) on prebaked quartz fiber filters (QFF; 90 mm, PALL Life Sciences). From July 24 to August 3, 2022, each sampler was equipped with a two-stage filter holder. In one sampler, the second stage placed behind the QFF contained a prebaked backup QFF to assess positive sampling artifacts due to gas adsorption. In the other sampler, the second stage was equipped with a precleaned polyurethane foam (PUF) filter (URG-2000–30–52PC) to collect gas-phase oVMS. QFFs were prebaked at 550 °C for 18 h. PUF filters were precleaned by sonication and repeated compressions using ultrapure water, acetonitrile and acetone, then air-dried.^32,37^ A field blank was collected for every five samples without active air flow.

### 2.3 D_5_ SOA from Laboratory Experiments

The environmental chamber setup and experimental methods have been previously described by Lewine et al.^30^ and the details specific to this study (from experiments E2-E4) are summarized in the Supporting Information, S1. Additional laboratory samples were generated from the oxidation of D_5_ using a 13 L oxidation flow reactor (OFR, Aerodyne Research, USA) operated in OFR185 mode as described elsewhere,^27,38^ with details specific to this study summarized in the Supporting Information, S2.

### 2.4 Sample Storage

All QFF samples from field, chamber, and OFR were stored in aluminum foil-lined, prebaked Petri dishes sealed with Teflon tape, while PUF samples from field, chamber, and OFR were transferred to prebaked (550 °C for 5.5 h) wide-mouth amber jars with Teflon-lined caps and sealed with Teflon tape. All samples were stored under dark conditions at −20 °C until extraction.

### 2.5 Organic and Elemental Carbon Analysis

Organic carbon (OC) and elemental carbon (EC) were measured on QFF containing ambient PM_2.5_ from NYC, chamber-derived SOA from D_5_, and OFR-derived SOA from D_5_ (all on 1.0 cm^2^ punches of QFF), and were quantified by thermal-optical analysis (Sunset Laboratory Inc.) following the NIOSH 5040 method (maximum He stage temperature: 870 °C), as described elsewhere.^39^

### 2.6 Analysis of oVMS

#### 2.6.1 Sample Preparation

Laboratory and field QFF samples were solvent extracted and analyzed by UPLC-MS/MS following the extraction procedure described by Meepage et al.^27^ Detailed extraction procedures are provided in the Supporting Information, S3. Finally, the extracts were reconstituted in 180 μL of a 95:5 acetonitrile-to-water extraction solvent, and 20 μL of D_5_-phenol internal standard (1000 μg L^–1^) was added, except in the case of NYC filters for which 11.6 cm^2^ of the filter was extracted when extracts were reconstituted in 90 μL, followed by addition of 10 μL of D_5_-phenol (1000 μg L^–1^) internal standard.

PUF samples were prepared for chemical analysis as described by Al-Naiema and Stone.^37^ Briefly, filters were extracted by three sequential compressions using acetonitrile and evaporated to 1 mL by rotary evaporation at 30 °C (120 rpm, 200 mbar). Then, the reduced extracts were filtered with PTFE filters (0.25 μm, Whatman, GE Health Care Life Sciences) and evaporated to 100 μL under a gentle stream of ultrapure nitrogen (99.999%, Linde) at 30 °C using the microscale nitrogen evaporation (Reacti-Vap I, Thermo Scientific). D_5_-phenol (1000 μg L^–1^) was added. A 1:20 dilution of the final extract was then prepared, with D_5_-phenol added as the internal standard.

#### 2.6.2 Instrumental Analysis by UPLC-MS/MS

oVMS were semiquantified using an ultraperformance liquid chromatograph (UPLC) coupled to a Q-Exactive Quadrupole Orbitrap mass spectrometer (Thermo Scientific) in negative mode with a heated electrospray ionization (ESI) source, following the procedure described by Meepage et al.^27^ Briefly, oVMS were separated using a reversed phase C18 column (2.1 mm × 100 mm, 1.7 μm particle size; ACQUITY Premier BEH C18 Waters) using a mobile phase of 10 mM ammonium bicarbonate buffered to pH 10 by adjustment with ammonium hydroxide. The aqueous portion of the mobile phase was held at 62% for 0.5 min, decreased to 15% over 10 min and held constant until 22 min before column re-equilibration. An ACQUITY Premier BEH C18 VanGuard FIT Cartridge (1.7 μm, 2.1 mm × 5 mm) was installed upstream of the analytical column for sample analysis. Consequently, retention times reported here are increased by approximately 0.2 min relative to those reported by Meepage et al.^27^

#### 2.6.3 Qualitative Analysis and Semiquantitation of oVMS

Qualitative analysis was performed as described in Meepage et al.^27^ and summarized in Supporting Information, S3. For semiquantitative analysis, the instrument was calibrated daily with a freshly prepared six-point calibration standard series of tris­(tert-butoxy)­silanol ranging 4–3785 nmol L^–1^. The lowest quantifiable signal corresponded to tris­(tert-butoxy)­silanol standard concentrations equivalent to 8 pg m^–3^ for the QFF and 14 pg m^–3^ for PUF. Data were acquired using Xcalibur 4.2 (Thermo Scientific) software. Quantification was performed using the software TraceFinder v5.0 (Thermo Scientific).

The analytical uncertainty in oVMS concentrations was calculated from the total relative uncertainty (%*e*_total_) propagated according to eq 1, accounting for the relative errors in air volume (%*e*_a_, 1%); extraction efficiency (%*e*_e_, 18%; which represents the difference in the observed and expected responses of spike recovery samples to which known amounts of analytes were added); the relative error of measurement (%*e*_m_; calculated from the limit of detection, 2 nmol L^–1^, and the measured concentration); and, if applicable, the relative error of dilution (%*e*_d_, 0.6%) propagated from measurement errors in initial and final volumes.

1 $$\%e_{t\mathrm{otal}}=\sqrt{{{\%e}_{a}}^{2}+{{\%e}_{e}}^{2}+{\%e_{m}}^{2}+{\%e_{d}}^{2}}$$

The overall analytical uncertainty does not include any bias introduced by semiquantitation against tris­(tert-butoxy)­silanol as a surrogate standard, since the extent of this bias can only be determined when oVMS are quantified against an authentic standard.

### 2.7 Data Analysis

#### 2.7.1 Positive Sampling Artifact

The current study extends prior measurements of positive sampling artifacts of oVMS derived from D_5_ by Welker et al.^32^ to include additional oVMS derived from D_4_ and D_6_. Briefly, positive sampling artifacts associated with oVMS for ambient aerosol samples were assessed for July 26 and 28 (*n* = 4). Positive sampling artifacts were calculated as the percent of oVMS on backup QFF filter (QFF_b_) relative to front QFF filter (QFF_f_) according to eq 2.

2 $$p\mathrm{ositive artifact}=\frac{{\mathrm{QFF}}_{b}}{{\mathrm{QFF}}_{f}}\times 100\%$$

#### 2.7.2 Gas Particle Distributions

The current study extends prior measurements of gas particle distribution of oVMS derived from D_5_ by Welker et al.^32^ to include additional oVMS derived from D_4_ and D_6_ for July 26 and 28 (*n* = 4). Briefly, the gas particle distributions of oVMS were calculated as the artifact-corrected particle phase percent *F*_p_, expressed as the percentage of oVMS in the particle phase relative to the total detected oVMS (eq 3).

3 $$F_{p}=\frac{{\mathrm{QFF}}_{f}-{\mathrm{QFF}}_{b}}{{\mathrm{QFF}}_{f}+\mathrm{PUF}}\times 100\%$$

The adsorbed gas-phase contribution was estimated as the amount detected on the QFF_b_, which adsorbs vapor passing through the QFF_f_. Particle phase oVMS were determined by subtracting the QFF_b_ amount from that collected on the front filter (QFF_f_). Gas-phase oVMS were determined by adding the QFF_b_ amount to that collected on the PUF.

#### 2.7.3 Statistical Analysis

Correlation coefficients, normality, and the significance of differences in daytime (D) and nighttime (N) samples were assessed using OriginPro, Version 2025b. (OriginLab Corporation, Northampton, MA, USA). Further details are provided in the Supporting Information, S6. Briefly, the normality of the natural log transformed concentrations were assessed using the Anderson–Darling test. Pearson correlation and Spearman correlation analysis were conducted on the log-transformed data. To evaluate differences between day and night samples, if both groups exhibited normal distributions after natural log transformation, a paired *t*-test was applied. If normality was not met but the data could be paired, a nonparametric Wilcoxon signed-rank test was used.

#### 2.7.4 Estimation of PM_2.5_ OC from VMS-Derived SOA in NYC

Estimates of VMS contributions to SOA focus on four samples collected over July 26 and 28, 2022 (*n* = 4), when gas and particle-phase concentrations were corrected for artifacts. The SOA tracer method for estimating SOA was applied, based upon oVMS to OC ratios observed in the chamber experiment of D_5_ oxidation.^40^ The selected chamber experiments (E2-E4 in Lewine et al.^30^) simulated atmospherically initiated oxidation of D_5_ by hydroxyl radicals at relatively low OH exposure (∼10^11^ molecules s cm^–3^) under specific RO_2_ fate conditions (where RO_2_ + HO_2_ was dominant ∼92%). These experiments represent early stage oxidation rich in siloxanol.^30^ The fraction of secondary organic carbon (SOC) mass derived from oxidation products of D_5_ (oD_5_) in the chamber experiment (*f*_oD5,chamber_ g gC^–1^) was determined as the ratio of the summed concentrations of oD_5_ in μg m^–3^ ([tracer_i_]) and total OC loading in (μgC m^–3^) on the same filter:

4 $$f_{\mathrm{oD}5,\mathrm{chamber}}={\left(\frac{\sum_{i}\left[{\mathrm{tracer}}_{i}\right]}{t\mathrm{otal OC loading}}\right)}_{\mathrm{chamber}}$$

The UPLC-MS/MS analysis of the chamber sample indicated that D_5_-derived siloxanol series dominated the product distribution under these conditions, in agreement with prior studies of D_5_ oxidation by OH.^30^ Because siloxanols appear to undergo further oxidation to disiloxanols and so on, the siloxanol series was summed when calculating *f*_oD5,chamber_.

The SOC derived from oD_5_ in NYC (ngC m^–3^) was then determined by dividing the summed ambient concentration of oD_5_ in ng m^–3^ ([tracer_i_]) by the *f*_oD5,chamber_ (g gC^–1^):

5 $$S{\mathrm{OC from oD}}_{5\;\mathrm{NYC}}=\frac{\sum_{i}{\left[{\mathrm{tracer}}_{i}\right]}_{\mathrm{NYC}}}{f_{\mathrm{oD}5,\mathrm{chamber}}}$$

Because NYC and chamber samples were both run under identical UPLC conditions and on the same day, any biases in semiquantitation are eliminated when their concentrations are ratioed in eq 5. The fraction of SOC from oD_5_ in NYC was then expressed as a percentage of the total OC loading (μgC m^–3^) measured using thermal-optical analysis. The contribution of D_5_-derived SOA mass to PM_2.5_ (μg m^–3^) was estimated using the carbon fraction of the disiloxanol (0.26), based on the average carbon fraction of siloxanols observed in NYC PM_2.5_ weighted by relative signal abundance (eq S1 in the Supporting Information). Artifacts are not expected to significantly influence *f*_oD5,chamber_, because adsorption artifacts are likely to be dominated by the siloxanol, which contributes to both the numerator and denominator of this fraction, while more oxidized oVMS are found to have minimal artifacts.^30^ Assuming equal ionization efficiencies for semiquantitation likely underestimates siloxanol concentrations in the NYC samples relative to the chamber experiments, making the estimated SOA contribution an upper-bound estimate. Due to the lack of chamber-derived mass fractions for D_4_ and D_6_, the *f*_oD5,chamber_ was used as a proxy to estimate SOC derived from oxidation products of D_4_ (oD_4_) and oxidation products of D_6_ (oD_6_) in NYC.

## 3 Results and Discussion

### 3.1 Qualitative Analysis of oVMS in New York City (NYC) and Average Signal Distributions

The most abundant oVMS signals detected by UPLC-MS/MS in NYC PM_2.5_ samples are summarized in Table 1 for July 8 through August 3, 2022. The relative strengths of oVMS signals depend upon their concentrations and ESI efficiency in the column eluent. Because authentic standards for the expected oVMS were unavailable at the time of this study, semiquantitation using tris­(tert-butoxy)­silanol as a surrogate standard was applied following Meepage et al.^27^ This approach assumes that all oVMS exhibit equivalent ionization efficiencies to the surrogate, which can introduce positive or negative biases in absolute concentrations. Consequently, the relative signal distributions reported in Table 1 should be interpreted as semiquantitative estimates, rather than measures of the relative abundance of these compounds in PM_2.5_.

**Table 1:**
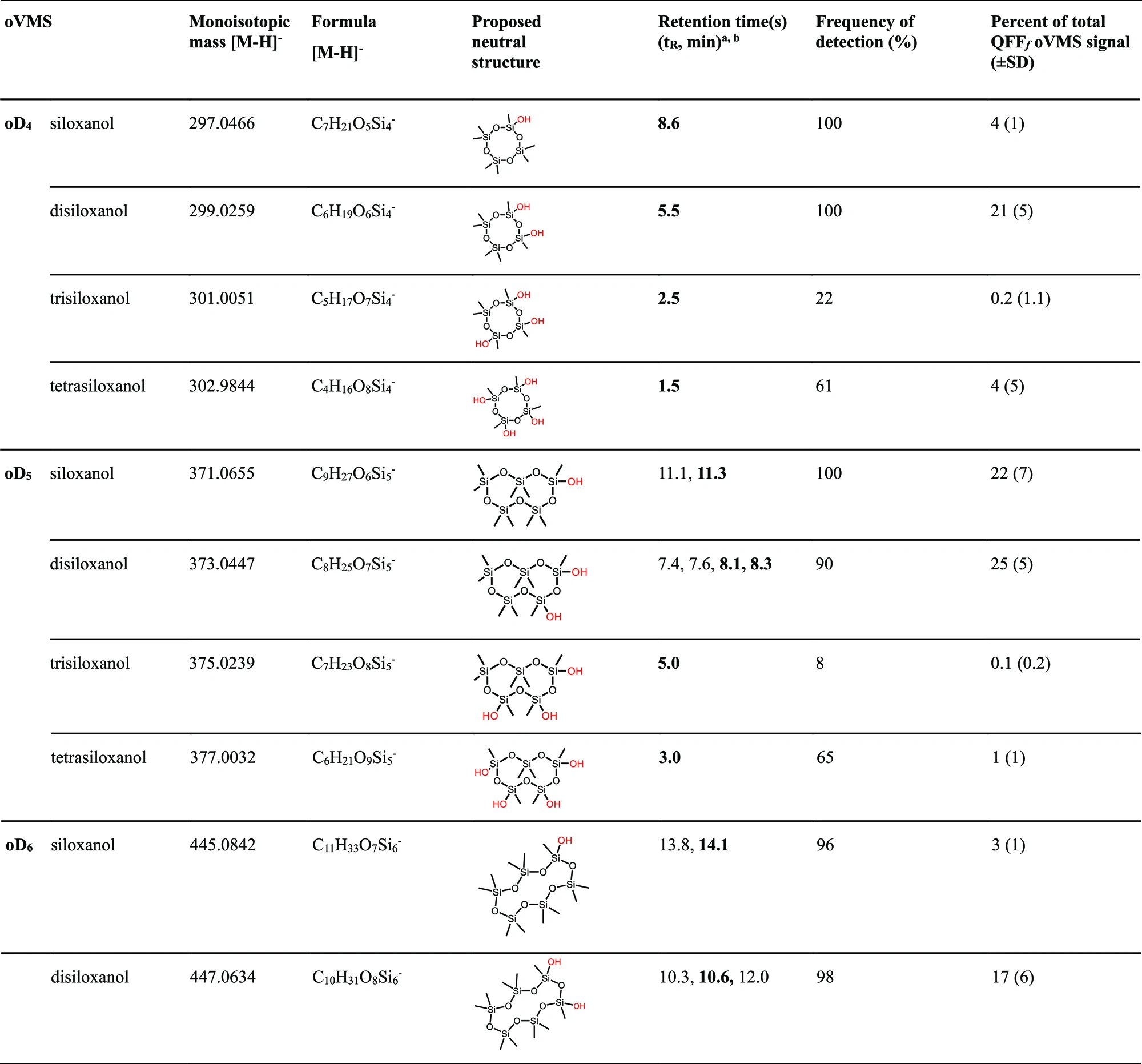
Major oVMS Signals Detected by UPLC-MS/MS in NYC PM_2.5_ Samples, Summarized with Their Expected Precursor VMS Based on the Si Number, Monoisotopic Mass [M-H]^−^, Chemical Formula, Proposed Structure, Retention Time/s (*t*_R_, min), Frequency of Detection, and Average Relative Signal Distribution Percent with One Standard Deviation (SD)

a In comparison to Meepage et al.,^27^ the retention times in this work are shifted by approximately 0.2 min for some of the compounds because of the use of the guard column at the start of the C_18_ column.

b The bolded retention times indicate the major oVMS signals (marked with stars in Figure S1).

The strongest oVMS signals detected in the PM_2.5_ samples were attributed to a series of siloxanols expected to be derived from VMS by replacement of one or more methyl groups with hydroxyl groups (Table 1 and Figure 1). Four members of the siloxanol series were observed for D_5_, including the siloxanol (C_9_H_27_O_6_Si_5_, *m*/*z* 371.0654), disiloxanol (C_8_H_25_O_7_Si_5_, *m*/*z* 373.0447), trisiloxanol (C_7_H_23_O_8_Si_5_, *m*/*z* 375.0239), and tetrasiloxanol (C_6_H_21_O_9_Si_5_, *m*/*z* 377.0032). This oxidation series is consistent with previous laboratory observations of D_5_ oxidation by OH radicals.^20,22,26,27^ The D_5_ siloxanol and disiloxanol were detected in every NYC sample and were the largest oVMS signals observed, with less frequent detection and lower concentrations of trisiloxanol (8%) and tetrasiloxanol (65%).

**Figure 1:**
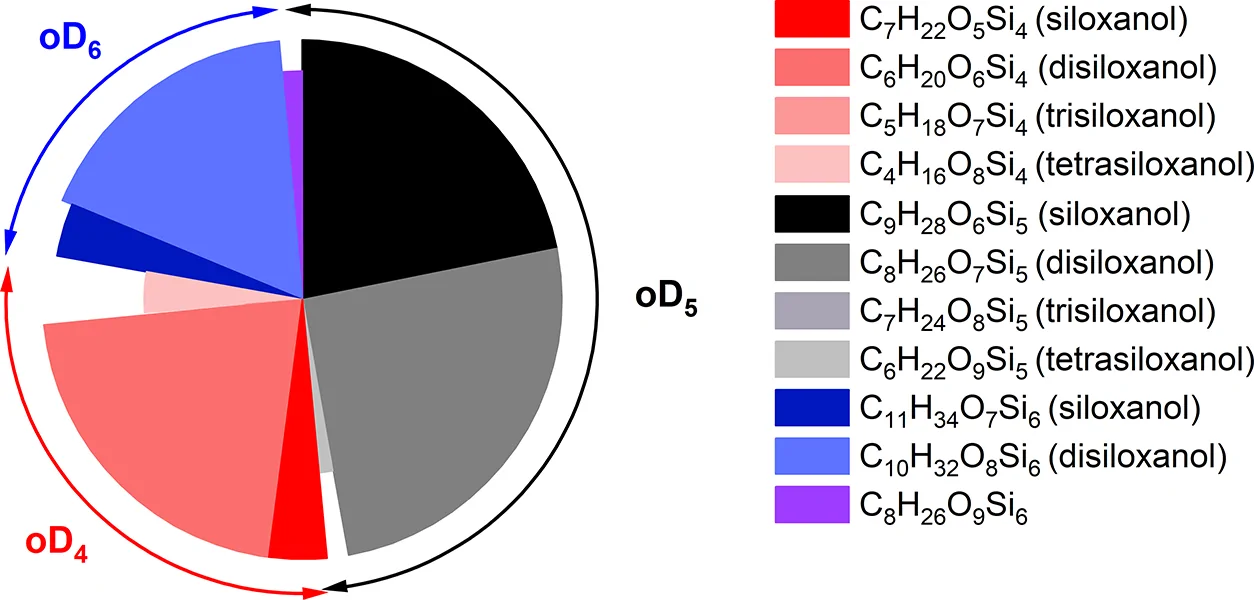
Major oVMS observed in PM_2.5_ at NYC-METS (July 8–August 3, 2022). The area of the pie chart shows average relative signal and radius of each pie shows detection frequency. D_4_ and D_5_ trisiloxanols are not visible due to their low abundance.

Analogous siloxanol series were observed for D_4_ and D_6_ in PM_2.5_ from NYC. D_4_-derived oVMS up to the tetrasiloxanol were detected, similar to previous laboratory oxidation of D_4_ by OH radicals.^20^ For D_6_-derived oVMS, up to two such steps were detected. The low abundance of the D_6_ precursor in the urban atmosphere likely limits the detectability of tri- and tetrasiloxanols.^7,41,42^ The signal abundance and frequency of detection of the D_4_ and D_6_ siloxanols place them among the most abundant oVMS signals detected in NYC PM_2.5_ (Table 1)_._ Many of the oD_4_–oD_6_ signals exhibited multiple chromatographic peaks that sometimes coeluted (Figure S1). Consistent with our prior study, MS/MS data and retention times demonstrated that the D_5_ disiloxanol was predominantly in the 1,2-isomeric configuration.^27^ When isomeric peaks were well resolved, they were integrated individually, in addition to the summed total, for subsequent data analysis. Figure S2 plots the retention times of the major oVMS signals listed in Table 1 against the number of silicon atoms in their molecular formulas, revealing a pattern in which the retention time on the C_18_ column increases linearly with each additional dimethyl–Si–O unit but decreases with each added hydroxyl group. These consistent retention time trends provided additional confidence in the assignment of the D_4_, D_5_, and D_6_ siloxanol series.

Qualitative analysis targeted additional oVMS reported previously in the literature.^20,27,30^ In addition to the siloxanols described above, *m*/*z* 433.0114 was consistently detected (frequency of 84%), which was assigned the formula C_8_H_26_O_9_Si_6_ (with two double bond equivalences). Two chromatographically resolved isomeric compounds were detected at *t*_R_ 8.9 and 9.2 min, accounting for 1% of the oVMS signal in QFF_f_ from July 8 to August 3. This molecular formula has been previously observed in laboratory and chamber oxidation studies of D_5_, suggesting it a minor oxidation product of D_5_; however, a D_6_ precursor cannot be excluded due to its molecular formula.^20,21^ oVMS derived from larger cyclic VMS homologues (oD_7_–oD_10_) were also screened in four QFF_f_ samples collected on July 26 and 28, 2022 and were not detected, with the exception of a small peak consistent with D_7_-derived disiloxanol in the July 28 QFF_f_ sample (RT 13.2 min) that was ∼100× lower than the D_5_-derived disiloxanol on the same filter. The near-absence of oD_7_–oD_10_ products indicates that D_4_–D_6_ are the dominant cyclic VMS precursors contributing to ambient oVMS in NYC.

### 3.2 Positive Sampling Artifacts and Gas Particle Distribution

Positive sampling artifacts associated with the major oVMS from D_4_ and D_6_ were assessed using day and night samples from July 26 and 28, 2022 (Figure 2). Results for D_5_ were previously reported, so the discussion here focuses on new measurements for D_4_ and D_6_, and how they relate to D_5_.^32^ All D_4_-derived siloxanol and disiloxanols were detected in all four backup samples (QFF_b_) indicative of gas sorption to QFF. For D_6_, siloxanol was not detected on QFF_b_, whereas disiloxanol was detected in three of four QFF_b_. D_4_ siloxanols exhibited the highest sampling artifacts, requiring significant correction, with an average artifact of 71% (Table 2), while D_4_ and D_6_-derived disiloxanols showed much smaller sampling artifacts in the range of 11–14%. Tetrasiloxanols from D_4_ were detected on QFF_f_, but not on QFF_b_, showing no appreciable sampling artifact. In QFF_f_, the D_6_ disiloxanol signal was five times higher than that of siloxanol, placing siloxanol on QFF_b_ below the method detection limit. Overall, these results are consistent with the sampling artifact behavior reported for D_5_ by Welker et al.^32^ indicating that particle phase siloxanols can be highly influenced by sampling artifacts requiring correction for accurate measurements, while such artifacts are much lower for disiloxanols.

**Table 2:** Siloxanol and Disiloxanols from D_4_, D_5_, and D_6_ Measured on July 26 and 28, 2022, Including Detection Frequency (*n* = 4), Average Sampling Artifacts (±SD), and Particle Phase Fractions (±SD) after Artifact Correction

					particle phase percent *F*_p_, %	
oVMS category		*t*_R_ (min)	frequency of detection in QFF_b_, %	positive artifact, % (SD)	average (SD)	range
oD_4_	siloxanol	8.6	100	71(20)	13(9)	1–20
	disiloxanol	5.5	100	14(9)	69(5)	64–75
oD_5_ ^a^	siloxanol	11.3	100	80(16)	10(8)	5–22
	disiloxanol	8.1 and 8.3	100	10(4)	77(5)	72–82
oD_6_	siloxanol	14.1	0	–^b^	–^b^	–^b^
	disiloxanol	10.6	75	11(16)	88(15)	71–100

a The positive sampling artifacts and particle phase percentages of major oD5 are previously reported in Welker et al.^32^

b “–” marks values below detection limits in QFF_b_ and PUF.

**Figure 2:**
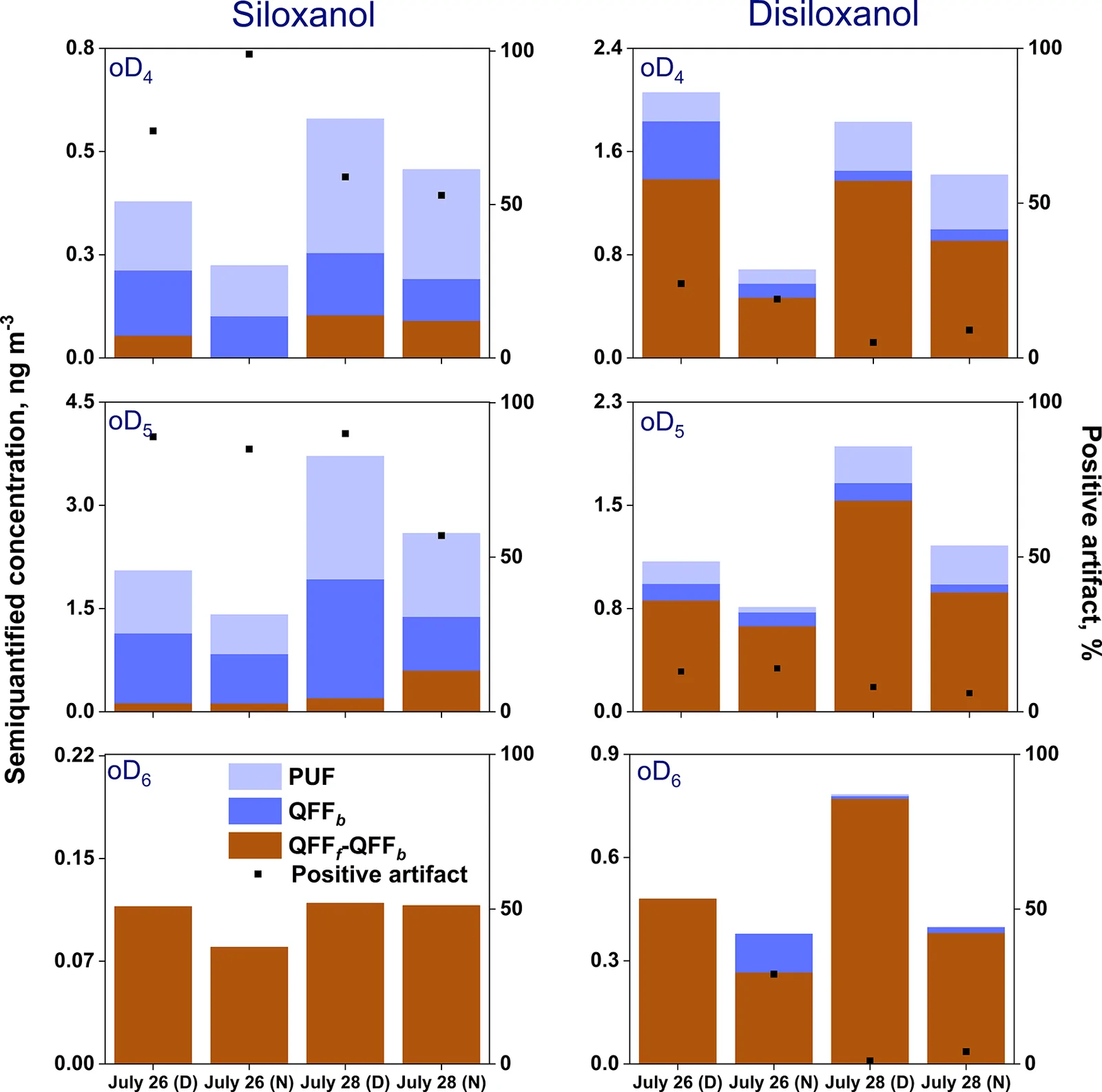
Semiquantified concentrations and gas particle distributions of oD_4_, oD_5_, and oD_6_ in NYC on July 26 and 28, 2022. The stacked bars show gas phase (PUF, light blue), adsorbed gas phase (QFF_b_, dark blue), and artifact-corrected particle phase (QFF_f_-QFF_b_, brown) concentrations in ng m^–3^. Black squares correspond to the right *y*-axis and indicate the positive sampling artifact (%) due to the adsorption of gas phase oVMS to QFF. Semiquantified particle phase concentrations of the tetrasiloxanols of D_4_ and D_5_ are presented in Figure S3 of the Supporting Information.

Gas particle distributions of oVMS from D_4_ and D_6_ were further assessed using the same four samples (Table 2), after artifact correction, and were compared with gas particle distributions of oD_5_ reported by Welker et al.^32^ D_4_ siloxanol resided predominantly in the gas phase, with <20% associated with particle phase. Disiloxanols from D_4_ and D_6_ were mainly associated with the particle phase with average particle fractions of 69 and 88%, respectively. Normalized signals corrected for extraction efficiency and sampling volumes demonstrated that more than 99% of D_4_-trisiloxanol and -tetrasiloxanol were in the particle phase. Overall, similar to D_5_, a progressive shift toward the particle phase was observed with increasing oxidation of D_4_, corresponding to lower volatility. D_6_ siloxanol was detected only in the particle phase, but its low abundance may bias the estimated particle fraction. Moreover, the lower volatility of oD_6_ due to the larger ring size likely also favors particle partitioning relative to the corresponding D_4_ and D_5_ products. Although the semivolatile D_5_ siloxanol is predominantly in the gas phase during summertime, this season is expected to be an annual minimum in particle partitioning based upon the relationship of its partition coefficient with temperature, while winter is expected to have the greatest partitioning to particles due to lower temperatures.^32^

### 3.3 Relative Ratios of oVMS Signals

#### 3.3.1 Relative Ratios of oVMS and VMS in NYC

The relative signals of total oVMS, inclusive of gas and particle phases, were compared to collocated VMS ratios from July 25 (N) to 28 (N), 2022 (*n* = 7, Table S1). Because the atmospheric half-lives of D_4_, D_5_, and D_6_ decrease with increasing ring size, larger-ring compounds are expected to be increasingly depleted during oxidation.^14^ Accordingly, VMS ratios (larger ring to smaller ring) are expected to be highest near emission sources and to decline during atmospheric transport as oxidation progresses.^42–44^ In NYC, D_5_/D_4_ and D_6_/D_5_ values averaged 3.3 and 0.05 for July 25 (N) to 28 (N), respectively.^7^ The D_5_/D_4_ ratio in NYC is consistent with the estimate of 3.0 for emission inventories associated with the general public use of VMS containing consumer products.^7^ In contrast, total oVMS (gas + particle) ratios measured in NYC were approximately 2.7 for oD_5_/oD_4_ and 0.2 for oD_6_/oD_5_. The oD_6_/oD_5_ ratio is significantly higher by a factor of 5 compared to the corresponding precursor ratio, consistent with the faster oxidation of D_6_. By contrast, the difference between the oD_5_/oD_4_ ratio and D_5_/D_4_ was not statistically significant, despite the higher oxidation rate of D_5_ relative to D_4_. Combining oVMS and VMS ratios in future studies could provide an additional observational constraint for evaluating the long-range transport of VMS.

#### 3.3.2 Relative Signal of Siloxanol and Disiloxanol as an Indication of the Extent of Oxidation

The ratio of the summed gas and particle concentrations of disiloxanols to total siloxanols serves as an indicator of the relative extent of VMS oxidation, reflecting the progression from first-generation (siloxanol) to more oxidized products (disiloxanol). This ratio was evaluated from July 25 (N) to July 28 (N) (*n* = 7). Because gas and particle phase concentrations were summed, the total oVMS concentrations are not affected by artifact formation. The average total disiloxanol to total siloxanol ratios were 3.8 (±1.2) for D_4_, 0.6 (±0.2) for D_5_, and 4.8 (±1.0) for D_6_ (Figure 3), indicating more extensive atmospheric aging of D_4_ and D_6_ compared to D_5_ in NYC, with more disiloxanol than siloxanol. The lower ratio observed for D_5_ suggests that it was predominantly in an earlier stage of oxidation, which is consistent with local D_5_ emissions in the urban airshed.^7^ In contrast, D_4_ and D_6_ likely have greater contributions from more distant sources (i.e., industrial emissions), allowing for more extensive atmospheric oxidation prior to detection.

**Figure 3:**
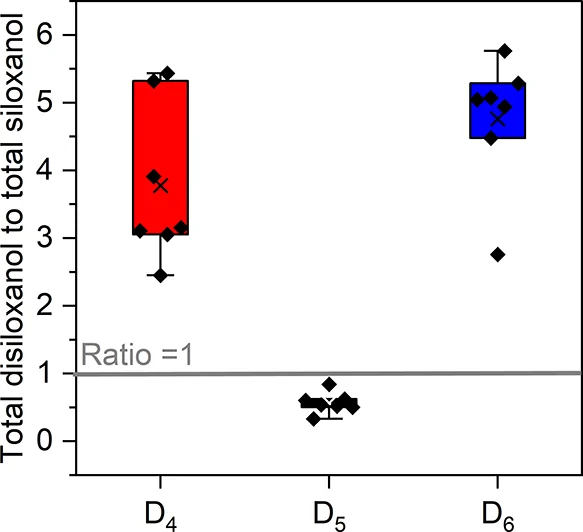
Total disiloxanol to total siloxanol ratio in NYC from July 25 (N) to July 28 (N), 2022 (*n* = 7) shown as box-and-whisker plots for D_4_, D_5_, and D_6_; black diamonds indicate individual data points.

#### 3.3.3 Comparison of Siloxanol and Disiloxanol Observed in the Field to Laboratory Studies

The relative abundances of oVMS in NYC are compared to those observed in laboratory studies of D_5_ oxidation, with the chamber being an example of early stage D_5_ oxidation (<1 day of atmospheric aging where RO_2_ + HO_2_ was dominant ∼92%) and the OFR being an example of late-stage oxidation (4 days equivalent aging where RO_2_ + HO_2_ was dominant ∼95%). To aid in comparison, gas- and particle-phases measurement were summed to eliminate the influence of sampling artifacts and oVMS responses were normalized to siloxanol (Figure 4). In the chamber, the siloxanol product dominated (97% of the total signal), with decreasing contributions from di-, tri-, tetra-, and pentasiloxanols. In the OFR, more oxidized products were observed, with di- and higher-order siloxanols accounting for half of the total siloxanol signal. In NYC samples, the D_5_ siloxanol product remained dominant, accounting for two-thirds of the oD_5_ signal, followed by disiloxanol. Tetrasiloxanols had only a very minor contribution (1%), and trisiloxanols were below quantification limits. Correspondingly, the observed disiloxanol to siloxanol ratios increased from 0.02 in chamber samples, to 0.6 ± 0.2 in NYC, and to 0.8 in the OFR. These ratios demonstrate that ambient air in NYC reflects more aging than the chamber, but less than the highly oxidative OFR. This finding is consistent with Lewine et al.^30^ who analyzed the same chamber samples and concluded that multiple OH oxidation steps are needed to form appreciable amounts of D_5_-derived SOA. Together, these observations suggest that D_5_ undergoes several days of atmospheric aging prior to detection as oD_5_ in NYC during summertime.

**Figure 4:**
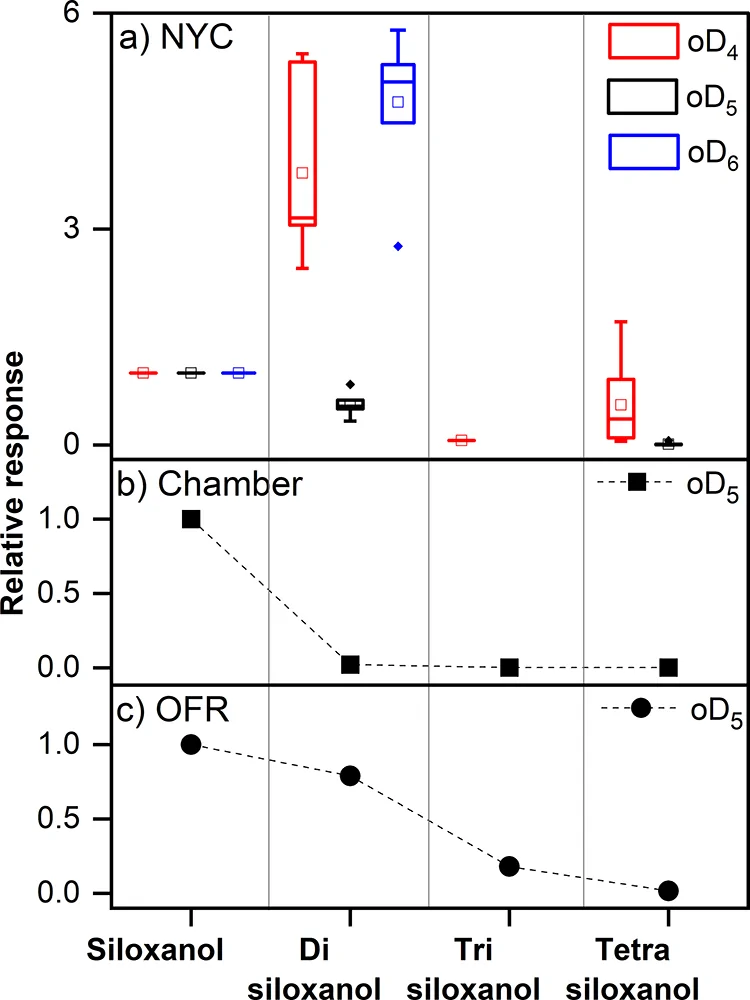
Gas and particle signals of oVMS normalized to the corresponding siloxanol signal in (a) NYC ambient PM_2.5_ from July 25 (N) to 28 (N), 2022 (*n* = 7, shown as box-and-whisker plots), (b) chamber samples from the reaction of D_5_ (<1 day atmospheric aging) and (c) OFR measurements from the reaction of D_5_ (4 days of atmospheric aging).

### 3.4 Summertime Trends in oVMS in NYC

#### 3.4.1 Temporal Trends

To assess temporal trends in ambient oVMS, PM_2.5_ measurements were extended to the full NYC time series from July 8 to August 3 (*n* = 51). Time series of oVMS concentrations are shown summed in Figure 5 and individually with uncertainties in Figure S4. These measurements have not been corrected for sampling artifacts; therefore, the concentrations measured on QFF include oVMS in the particle phase and gases adsorbed to QFF as discussed in Section 3.2.

**Figure 5:**
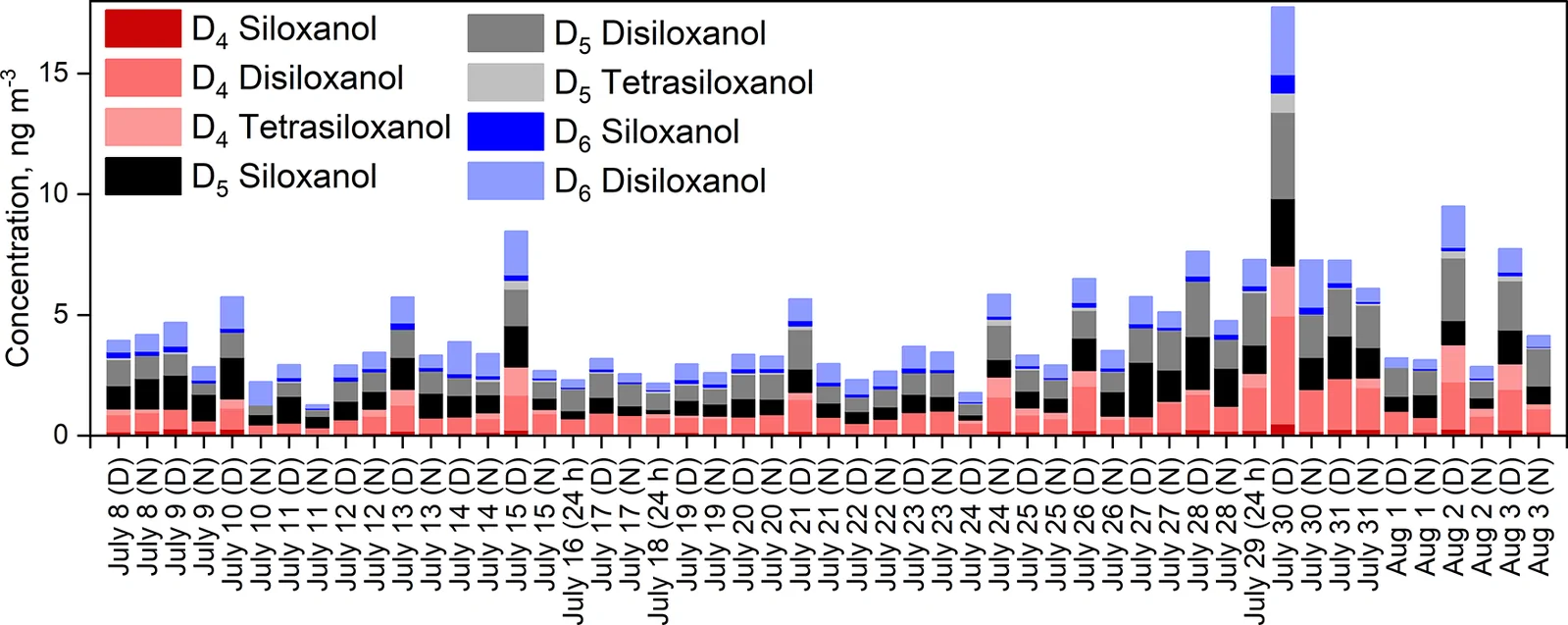
Time series of oD_4_, oD_5_, and oD_6_ siloxanols in NYC PM_2.5_ (without artifact correction), from July 8 to August 3, 2022, semiquantified against tris­(tert-butoxy)­silanol.

oVMS retaining the D_4_, D_5_, and D_6_ backbone were consistently detected throughout the sampling period, with high frequency of detection for siloxanols and disiloxanols (100%), but much lower detection of trisiloxanol (<22%) and tetrasiloxanols (<66%). The D_4_, D_5_, and D_6_-derived siloxanols and disiloxanols exhibited high variability as indicated by the coefficient of variation (CV = 52–69%, calculated with nonzero values), with even greater variability for trisiloxanols (46–212%) and tetrasiloxanols (116–144%). The high detection frequency indicates the sustained presence of oVMS during summertime, with time-varying concentrations.

The highest PM_2.5_ oVMS concentrations were observed on July 30 (D), when siloxanol levels reached 3 to 5 times the campaign average. On this day, OC loading was the lowest of the sampling period and approximately two times lower than the campaign average. Meteorological conditions were characterized by high wind speeds (90th percentile) originating from urban areas to the northwest, low relative humidity (40.5%, below the fifth percentile), which favors adsorption to particles, and slightly elevated temperatures (1.0 °C above the campaign average, Figure S5).^7,17,36^ According to Welker et al.,^32^ the majority of D_5_ siloxanol observed in the particle phase in NYC was attributed to adsorption by the Junge-Pankow partitioning model, with negligible contributions from absorption. During a documented heat wave from July 20 to 24, when daily maximum temperatures reached approximately 33–35 °C, neither oVMS concentrations nor VMS precursor concentrations differed significantly from the campaign averages, although total organic aerosol mass increased by 42%.^7,36^ The lowest concentrations of many siloxanols occurred on the rainiest day (18 July, 24-h sample), whereas disiloxanols remained within the second to third quartile of the data set. Siloxanols primarily partition to the gas phase, while disiloxanols preferentially partition to the particle phase, which suggests different removal processes for the two species. Overall, these results indicate that summertime variability in oVMS concentrations in NYC is influenced by chemical aging, gas particle partitioning, and meteorology.

#### 3.4.2 Diurnal Trends

Diurnal trends in oVMS concentrations were examined through day/nighttime samples with and without normalization for planetary boundary layer height. In the unadjusted data, statistically significant differences (*p* < 0.05) were observed between daytime and nighttime concentrations of D_5_ siloxanol and D_5_ disiloxanol, with higher concentrations occurring at daytime (Figure S6). After multiplying oVMS concentrations by the boundary layer height, the day–night contrast remained statistically significant (*p* < 0.05), with higher boundary layer height-adjusted concentrations observed during the daytime (Figure S7). This elevated daytime concentration is primarily driven by the enhanced daytime photochemical activity, and it drives greater formation of secondary oVMS products during daytime.^7,45^ A similar behavior was observed for oD_4_ and oD_6_, supporting a consistent pattern across precursors. Overall, these results are consistent with previous observations of higher nighttime precursor concentrations and daytime oxidation based on observations of VMS.^7^

#### 3.4.3 Correlations

Correlation analysis was applied to evaluate whether oVMS share common formation pathways and whether local precursor enhancements translate into oxidized products within the urban airshed (see S6.2, for detailed description). Only oVMS with >92% detection frequency, specifically siloxanol and disiloxanols, were included. Correlation strength was classified as very high correlation (*r* = 0.9–1.0), high (0.7–0.9), moderate (0.5–0.7), low (0.3–0.5), or negligible (0.0–0.3).^46^ Correlations were first examined between siloxanol and disiloxanol signals at different retention times (*t*_R_) for D_5_ and D_6_, while D_4_ oVMS were detected at only one *t*_R_. For subsequent analyses, siloxanol and disiloxanol signals at different *t*_R_ were summed separately for siloxanol and disiloxanol to enable direct comparison. Relationships between the summed siloxanol and disiloxanol were then evaluated, followed by their correlations with corresponding parent precursors. Finally, the associations with environmental variables (temperature, wind speed, NO_*x*_, ozone, relative humidity, precipitation accumulation, and organic carbon loading), were examined. Together, these analyses aim to distinguish the relative influence of local precursor availability, chemical processes and environmental variables in governing oVMS.

The correlation coefficients were moderate to high for the siloxanol and the disiloxanol detected at different *t*_R_ values for D_5_ and D_6_ (Figures S8 and S9). Moderate to high correlations were also observed among siloxanol and disiloxanols, within and across D_4_, D_5_, and D_6_ precursors, further suggesting that these oVMS share common atmospheric formation pathways. In contrast, negligible correlations were observed between siloxanols and their precursor VMS, and only low correlations were observed between disiloxanols and their precursor VMS. Siloxanol and disiloxanol concentrations did not increase on days with local VMS emission spikes, indicating that local precursor enhancements do not immediately generate local oVMS.^7^ This is consistent with the atmospheric lifetime of VMS, which varied from 3 to 10 days, such that VMS would be converted to oVMS far downwind of the urban areas where they are released.^14,15^ Moreover, siloxanols and disiloxanols did not correlate consistently with environmental variables, reflecting their complex behavior and relatively long lifetimes. Overall, these results indicate that oVMS from D_4_, D_5_, and D_6_ follow similar oxidation pathways over days to week-long time scales.

### 3.5 Estimation of VMS Contributions to SOA Using the SOA Tracer Approach

To apportion PM_2.5_ to SOA derived from VMS, secondary organic carbon (SOC) from oD_5_ was estimated using eq 5. SOC was subsequently converted to SOA concentrations by the carbon fraction of the D_5_-derived SOA, estimated as the carbon fraction of the D_5_ disiloxanol (0.26), which was the most abundant oVMS in PM_2.5_ in NYC after artifact correction (Figure 2). The SOC derived from D_5_ was then expressed as a percentage of total PM_2.5_ OC (Figure 6). Due to the lack of chamber-derived mass fractions for D_4_ and D_6_, the D_5_ mass fraction was used as a proxy. Across the four PM_2.5_ samples with artifact correction (July 26 and July 28, 2022), the estimates of D_4_, D_5_, and D_6_-derived SOA are summarized in Table 3 and accounted for a total SOA concentration of 13.7 ± 4.5 ng m^–3^. The SOC summed over oD_4_, oD_5_, and oD_6_ was estimated to be 3.5 ± 1.1 ngC m^–3^ and had a very small contribution to total OC in PM_2.5_ in NYC (0.10 ± 0.05%).

**Figure 6:**
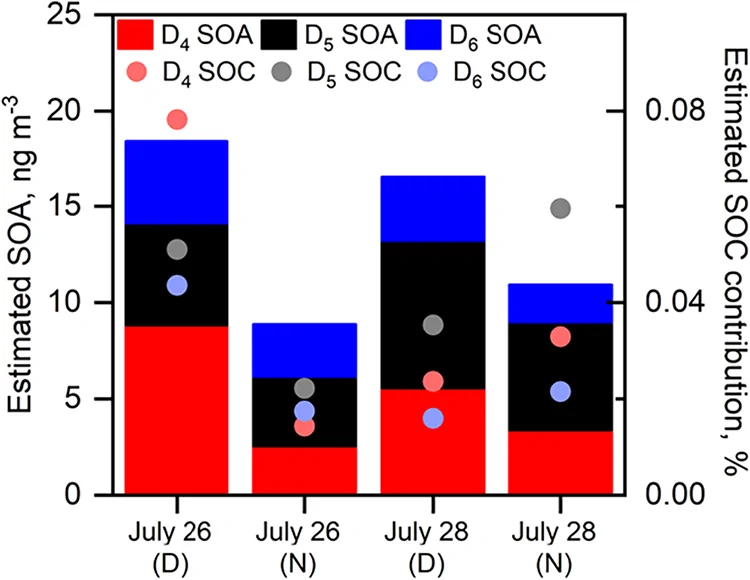
Estimated VMS-derived SOA concentrations (ng m^–3^, left axis) and percent contributions of oVMS-derived SOC to OC (% OC, right axis) from D_4_, D_5_, and D_6_ during daytime (D) and nighttime (N) periods on July 26 and July 28, 2022.

**Table 3:** Average Estimates of PM_2.5_ SOA, SOC, and Percent Contribution of D_4_, D_5_, and D_6_-Derived SOC to OC (± One Standard Deviation) in NYC^a^

	July 26 and July 28, 2022 average (with paired artifact correction, *n* = 4)			July 19 to August 3, 2022 average (with average artifact correction, *n* = 31)		
precursor	oVMS SOA (ng m^–3^)	oVMS SOC (ngC m^–3^)	oVMS OC (%)	oVMS SOA (ng m^–3^)	oVMS SOC (ngC m^–3^)	oVMS OC (%)
D_4_	5.1 (2.4)	1.2 (0.7)	0.04 (0.03)	7.0 (5.6)	1.7 (1.4)	0.04 (0.05)
D_5_	5.5 (1.7)	1.4 (0.4)	0.04 (0.02)	7.4 (4.0)	1.9 (1.1)	0.05 (0.04)
D_6_	3.1 (1.0)	0.8 (0.3)	0.02 (0.01)	4.0 (2.4)	1.1 (0.6)	0.03 (0.03)
sum	13.7 (4.5)	3.5 (1.1)	0.10 (0.05)	18.4 (11.6)	4.7 (2.9)	0.12 (0.12)

a Results are shown for July 26 and July 28 (samples with backup filters) and for July 19 to August 3 (artifact-corrected using average values).

To extend this estimation to NYC samples collected from July 19 to August 3 (*n* = 31), the average artifact for each oVMS determined by eq 2 for four samples with backup filters was applied to the larger data set to estimate artifact-corrected SOA concentrations. The application of the average artifact correction was not expected to introduce substantial error, based on the 26 and 28 July 2022 samples having no significant differences between the paired and averaged artifact corrections (Table S5). For the extended data set, D_4_, D_5_, and D_6_-derived SOA were estimated to account for an average of 18.4 ± 11.6 ng m^–3^. This corresponds to 0.17 ± 0.16% of PM_2.5_ mass, where PM_2.5_ mass was estimated from measured OC concentrations using an average PM_2.5_ OC mass fraction of 0.36, calculated from PM composition observations at a regulatory PM monitoring site in the Bronx (NYC IS 52)^47^ as described in Section S7 of the Supporting Information. In terms of OC, the VMS-derived SOA contribution for the extended data set corresponded to 4.7 ± 2.9 ngC m^–3^ and accounted for 0.12% of total PM_2.5_ OC. Overall, the extended sampling period (*n* = 31) results consistently demonstrate that D_4_, D_5_, and D_6_-derived SOA contribute very minimally to total OC in PM_2.5_ in NYC.

Uncertainties in the apportionment of SOC and PM_2.5_ to VMS-derived SOA result from assumptions about the applicability of chamber-based measurements to NYC. The ratio *f*_oD5,chamber_ does not fully reflect the environmental conditions in NYC, with expected differences in precursor concentrations, oxidant levels, particle composition, and environmental conditions like temperature and humidity.^17,30^ As discussed in Section 3.3.3, product distributions under different OH exposure conditions suggest that D_5_ requires several days of atmospheric aging to achieve the levels of oxidation observed in NYC. In contrast, the chamber experiments conducted in this study represent less than 1 day of atmospheric aging. As a sensitivity analysis, the fraction of SOC mass derived from oVMS was determined by using chamber and OFR experiment data for ambient NYC measurements on 26 and 28 July 2022 (*n* = 4). The chamber and OFR samples were analyzed under identical UPLC conditions, although they were analyzed on a different day from the NYC sample. The SOC mass estimated from oVMS was approximately a factor of 2 lower when using the OFR-derived *f*_oD5_ (for approximately 4 days of atmospheric aging) compared to the chamber result (Table S6). This result suggests that our chamber-based *f*_oD5_ values are upper limits based on the available data and that relative uncertainties are large. Additionally, our use of *f*_oD5,chamber_ ratios derived from D_5_ chamber experiments is not expected to accurately represent the individual behavior of D_4_ and D_6_. On the basis of the average reaction rate constants of D_4_, D_5_, and D_6_,^15^ we approximate that D_6_-derived SOA is overestimated by 8% relative to D_5_ whereas the D_4_-derived SOA is underestimated by 27%.^15^

Our estimates of SOA from D_4_, D_5_, and D_6_ using the SOA tracer approach generally agree with prior field-based estimates of Si-SOA reported by Xu et al.^33^ Their study estimated Si-SOA as water-soluble silicon in PM_2.5_ over Beijing in August 2018, reporting an average concentration of 12.7 ± 10.2 ng m^–3^ that was interpreted to be from VMS. This Si-SOA contributed 0.06 ± 0.07% of PM_2.5_ mass in Beijing on average, with peak values reaching 0.26% on some days.^33^ Our observational estimates, specific to D_4_, D_5_, and D_6_, are of the same order of magnitude in terms of average concentrations and closely match the SOA contribution to total PM_2.5_ mass (0.17 ± 0.16%) for the period of July 19 to August 3, 2022, also reaching a maximum of 0.90% on July 30 (D). Together, these comparisons suggest that VMS oxidation represents a minor, but consistent source of urban SOA in megacities.

For D_5_, our observational-based estimates of oVMS are similar in magnitude to prior modeling studies. Our average oD_5_ (gas + particle) on July 26 and 28 (*n* = 4) was 14.6 ng m^–3^ which is within a factor of 4 of CMAQ modeling results from Janechek et al.^19^ who estimated an average of 4.6 ng m^–3^ of oD_5_ in NYC for July 2011 VMS emissions. Our particle phase measurements are also within a factor of 2 of the estimated D_5_-derived SOA (3.2 ng m^–3^) using an empirical model constrained by maximum observed VMS concentrations in Chicago for August 2011, which utilized high-NO*_x_* SOA yields from seeded OFR experiments, and an assumed OH exposure equivalent to approximately 0.63 atmospheric days.^24^ Although their study did not include NYC, both Chicago and NYC are densely populated urban centers with high VMS emissions. More broadly, our observational-based estimates of VMS derived SOA are also similar in magnitude to the siloxane- and silane-derived SOA concentration modeled in Pasadena, California, in 2010.^34^ Overall, these comparisons support the conclusion that our observation-based oD_5_ and SOA estimates are broadly consistent with prior model-based assessments.

For D_4_ and D_6_, our observational-based compound-specific oVMS estimates are substantially higher than the prior modeling studies. Our average oD_4_ and oD_6_ (gas + particle) on July 26 and 28 (*n* = 4) was 8.0 and 3.3 ng m^–3^, respectively. Janechek et al.^19^ reported average concentrations of 0.8 and 0.3 ng m^–3^ for oD_4_ and oD_6_, respectively, whereas our measurements exceed these results by more than an order of magnitude. Han et al.^24^ predicted a maximum D_4_-derived SOA concentration of 0.04 ng m^–3^ and no D_6_-derived SOA due to the zero yield observed under experimental conditions, whereas our measurements exceed these results by at least 2 orders of magnitude. These greater differences in measurements and models observed for D_4_ and D_6_ compared to D_5_ suggest a greater relative abundance of oVMS from D_4_ and D_6_ than what has been predicted by models.^19,24^ Some of this difference is expected to arise from semiquantification, including differences in ionization efficiency across oVMS, and from the use of *f*_oD5_ as a proxy for oD_4_ and oD_6_. Additionally, the HENRYWIN-predicted oVMS Henry’s law constants used by Janechek et al.^19^ were later found to be approximately 50 to 150 times too high after specific updates for organosilicon compounds were made.^19,48^ It is possible that these constants inflated dry deposition velocities and thus reduced modeled atmospheric oVMS concentrations, with the largest bias for oD_6_ and the smallest for oD_4_. Uncertainty in emissions inventories for D_4_ and D_6_, contributions from the Cl oxidation pathway,^14,48^ and limitations in modeling removal of oD_5_ from the atmosphere via chemical and depositional processes may also contribute to these discrepancies. These results suggest that D_4_ and D_6_ remain the largest uncertainty in current oVMS SOA estimates and that these VMS should be a focus of future laboratory experiments of SOA formation, given their relatively large signals compared to D_5_ and sparse experimental characterization.

Although VMS oxidation is predicted to be a minor contributor to ambient PM_2.5_ in NYC summertime 2022, its relative importance is expected to increase during winter when lower temperatures enhance gas-to-particle partitioning^17,32,33^ and in regions downwind of major urban areas following oxidation of VMS to oVMS.^19^ As transportation and industrial emissions of VOC decline, volatile chemical products like VMS are expected to become an increasingly important source of SOA precursors.^12^ The SOA tracer approach presented herein provides a means to evaluate the impact of VMS on SOA under urban and other impacted regions in the future. Further improvement to the apportionment of SOA to VMS and other volatile chemical products necessitates further development of SOA standards, correction for sampling artifacts, and quantitative analysis of oxidation products from important SOA-forming precursors.

## Supplementary Material



## Data Availability

The data underlying this study is available at Iowa Research Online (doi: 10.25820/data.008439).

## Notes

Experimental details for chamber and oxidative flow reactor studies, qualitative oVMS analysis, QFF sample preparation, figures of extracted chromatogram tracers, retention time trends, time series, meteorological data, comparison of oVMS and parent VMS ratios, description of statistical analysis, results for day night differences, boundary layer height-adjusted day night differences, normality testing, and correlation analysis (PDF)
