# Supplementary material for: Tracking Oxidized Volatile Methyl Siloxanes in New York City in Summertime
Source: ACS EST Air. 2026 Sep 3;3(10):2591–603. doi: 10.1021/acsestair.6c00134 (PMC13660350; doi:10.1021/acsestair.6c00134)
Supplement: Supplementary file 1 [file ea6c00134_si_001.pdf]

Supporting Information

Tracking Oxidized Volatile Methyl Siloxanes in New York City in Summertime

Jeewani N. Meepage[1], Josie K. Welker[1], Christopher E. Brunet[2], Charles O. Stanier[3], Elizabeth A. Stone[1,3]

[1]University of Iowa, Department of Chemistry, Iowa City, Iowa 52242 USA

[2]University of Iowa, Department of Civil and Environmental Engineering, IIHR-Hydroscience & Engineering, Iowa City, Iowa 52242 USA

[3]University of Iowa, Department of Chemical and Biochemical Engineering, Iowa City, Iowa 52242 USA

Number of pages: 17

Number of supplementary information sections: 7

Number of figures: 9

Number of tables: 6

S1: D_5 SOA from chamber

The reaction chamber is a 20 m^3 fluoroethylene propylene (FEP) Teflon bag, with photolysis initiated using blacklights. To promote SOA formation, ammonium sulfate was introduced by atomizing a 1 or 2 g L^{-1} solution. Once the chamber reached the desired aerosol surface area (390–880 μm^2 cm^{-3}), precursor D_5 (52–76 ppb_v) and hydrogen peroxide (~30 ppm) were sequentially added. Following injection, the chamber was filled to its full volume and allowed to equilibrate for 30-45 minutes before the blacklights were turned on. Oxidation proceeded for 2–5 hours, after which the lights were turned off, and air samples were collected over 3 hours at a flow rate of 20 L min^{-1} using a two-stage filter holder. In the first stage, particulate matter was captured on pre-cleaned QFF (47 mm, PALL Life Sciences; baked at 550 °C for 18 hours). Semi-volatile gases were collected in the second stage using pre-cleaned PUF filters (URG-2000-30). The chamber pressure was maintained at ambient laboratory conditions (~860 mbar) by lowering the chamber bag volume during the sample collection. Under these conditions, the $RO_2 + HO_2$ reaction pathway was estimated to account for ~92% of the radical fate, while $RO_2 + NO$ contributed 6.1%. Isomerization occurred 1.4% of the time, $RO_2 + OH$ accounted for 0.4%, and 0.1% was $RO_2 + RO_2$ reactions.[1] Full descriptions of these experiments are provided in Lewine et al.[1]

S2: D_5 SOA from oxidative flow reactor

The OFR was used here to simulate several days of atmospheric aging under steady-state experimental conditions, enabling the formation of gas- and particle-phase samples within a short experimental timescale. This study focuses on an experiment that simulated 4 days of atmospheric aging, based on a 24-hour average OH concentration of 1.2×10^6 molecules cm^{-3}. Clean, dry air was introduced at a flow rate of 4.5 L min^{-1}, resulting in a mean residence time of 177 seconds. Air samples were collected downstream at a flow rate of 3.6 L min^{-1} using a two-stage filter holder. Particulate matter was collected in the first stage on pre-cleaned QFF (47 mm, PALL Life Sciences) baked at 550 °C for 18 hours. Semi-volatile organic compounds were captured in the second stage using pre-cleaned PUF (URG-2000-30). The experiment was performed at 28 ± 1 °C and 5% relative humidity. To control RO_2 fate, 43.3 ppm CO was added alongside the precursor D_5, resulting in an estimated $RO_2 + OH$ contribution of approximately 5%, with the remaining ~95% attributed to $RO_2 + HO_2$ reactions.[2] Full descriptions of these experiments are provided in Mohammadi et al.[2]

S3: Qualitative analysis of oVMS

Qualitative analysis was performed with Compound Discoverer 3.3.0 (Thermo Scientific). Initially, peaks detected in the field blanks were identified and subtracted. A targeted analysis was developed using 135 mass-to-charge ratios (*m/z*) previously observed in SOA samples generated from oxidation of D_5, extracted and directly analyzed by ESI Q-Exactive Quadrupole Orbitrap mass spectrometer.[3,4] An additional targeted analysis was applied to account for anticipated structural modifications, such as the substitution of methyl groups with OH or CH_2OH, allowing for up to 10 replacements. Peaks were assigned molecular formulas based on the following criteria: (a) elemental composition falling within the ranges of $C_{0-30}H_{0-90}O_{0-30}Si_{0-}$

$_{20}N_{0-10}S_{0-5}$, (b) theoretical *m/z* values within ±5 ppm of the observed m/z, and c) agreement between observed and theoretical isotopic patterns (A, A+1, and A+2 peaks), calculated for each candidate formula based on its number of silicon atoms, with a minimum of 50%. The characteristic isotopic pattern arising from the natural abundances of silicon isotopes ^{28}Si (92.2%), ^{29}Si (4.7%), and ^{30}Si (3.1%) provides a distinctive mass spectral signature that aids in the identification of oVMS compounds.

S4: QFF sample preparation

Extracted filter portions were either 11.6 cm^2 or 23.0 cm^2 of sampled area for ambient NYC $PM_{2.5}$ samples, 0.12 cm^2 for chamber PM samples, and 4.00 cm^2 for OFR PM samples. Filters were extracted twice sequentially by sonication (30 min each, 60 sonics min^{-1}) with acetonitrile and ultra-pure water (95: 5, 10 mL) and then filtered through polypropylene membrane syringe filters (0.45 µm followed by 0.20 µm pore size, PuradiscTM 25PP, Whatman®). The resulting volume reduced to 500 µL under a stream of ultra-high purity nitrogen gas (≤ 5 psi) at 50 °C using an evaporation system (Turbovap® LV, Caliper Life Sciences). Extracts were transferred to LC vials (1.5 mL, Agilent) and evaporated nearly to dryness under a very light stream of ultra-high purity nitrogen gas at 50 °C using a microscale nitrogen evaporation system (Reacti-Therm III TS 18824 and Reacti-Vap I 18825, Thermo Scientific).

S5: Average C fraction of oVMS in NYC $PM_{2.5}$

The average carbon fraction of oD_5 in NYC $PM_{2.5}$ was calculated using the following equation.

$$\text{Average C fraction of oVMS in NYC } PM_{2.5} = \frac{\sum_i A_i f_i}{\sum_i A_i}, \qquad \text{Equation S1}$$

where A_i is the concentration of each oVMS species and f_i is the carbon mass fraction of each oVMS, calculated from its molecular formula. The D_5-derived siloxanol series, including siloxanol, disiloxanol, and tetrasiloxanol, was used to represent oD_5. For the artifact-corrected samples collected on July 26 and 28, 2022 (n = 4), the concentration-weighted average carbon mass fraction of oVMS in NYC was calculated as 0.26, which is approximately equal to the carbon mass fraction of the D_5 disiloxanol.

S6: Data analysis

S6.1. Day vs night significance test

To evaluate differences between day and night samples, the data were first natural log–transformed. Normality of the natural log-transformed variables was assessed separately for day and night datasets using the Anderson–Darling test at a 95% confidence interval. For each variable, comparisons between day and night were then conducted. If both groups exhibited normal distributions and sufficient paired data were available, a paired *t*-test was applied. If normality was not met but the data could be paired, a nonparametric Wilcoxon signed-rank test was performed. The results are summarized in Table S2 and Figure S6. To evaluate the

differences in the boundary layer height (BLH)-adjusted day–night samples, the same steps described above were applied to the BLH-adjusted data, and the results are summarized in Table S3 and Figure S7.

S6.2. Correlation analysis

The Anderson–Darling normality test was first performed on the original dataset. The data were then converted to natural logarithmic form, and the normality test was repeated. Variables with an Anderson–Darling statistic less than the critical value at $\alpha = 0.05$ were considered to follow a normal distribution at the 95% confidence level (Table S4). In most cases, normality was achieved after log transformation. Therefore, Pearson correlation analysis was conducted on the log-transformed data, and the results are summarized in Figure S8. Since some variables did not achieve normality even after transformation, Spearman correlation analysis was also performed on the log-transformed data, with results shown in Figure S9.

S7: OC to $PM_{2.5}$ fraction: Measurements of $PM_{2.5}$ mass and $PM_{2.5}$ OC collected in the Bronx, New York City, at the NYC Intermediate School 52 monitoring site via the EPA Air Quality Data Mart and were used to determine the OC fraction of $PM_{2.5}$ mass.[5] In this analysis, $PM_{2.5}$ samples were collected onto filters over 24-hours every third day, mass was determined gravimetrically, and OC was determined by thermal-optical analysis. Based on measurements between July 7 and August 3, 2022, the average $PM_{2.5}$ OC fraction was calculated to be 0.36.

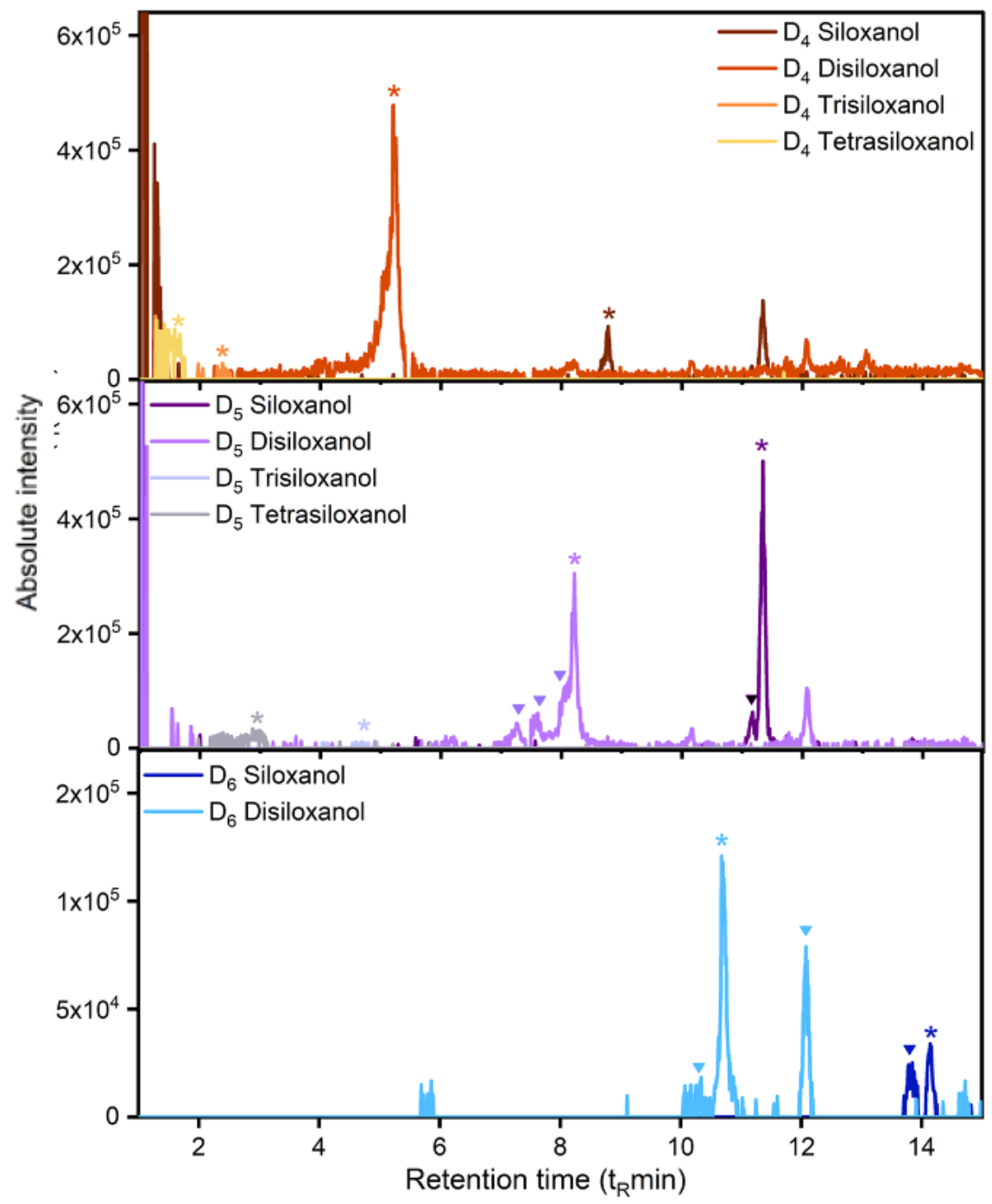

Figure S1: Extracted chromatograms of the oVMS signals listed in Table 1 for a $PM_{2.5}$ sample collected on July 15, 2022 during the daytime. The retention times of the major oVMS signals are marked with stars, and minor oVMS signals are labeled with triangles.

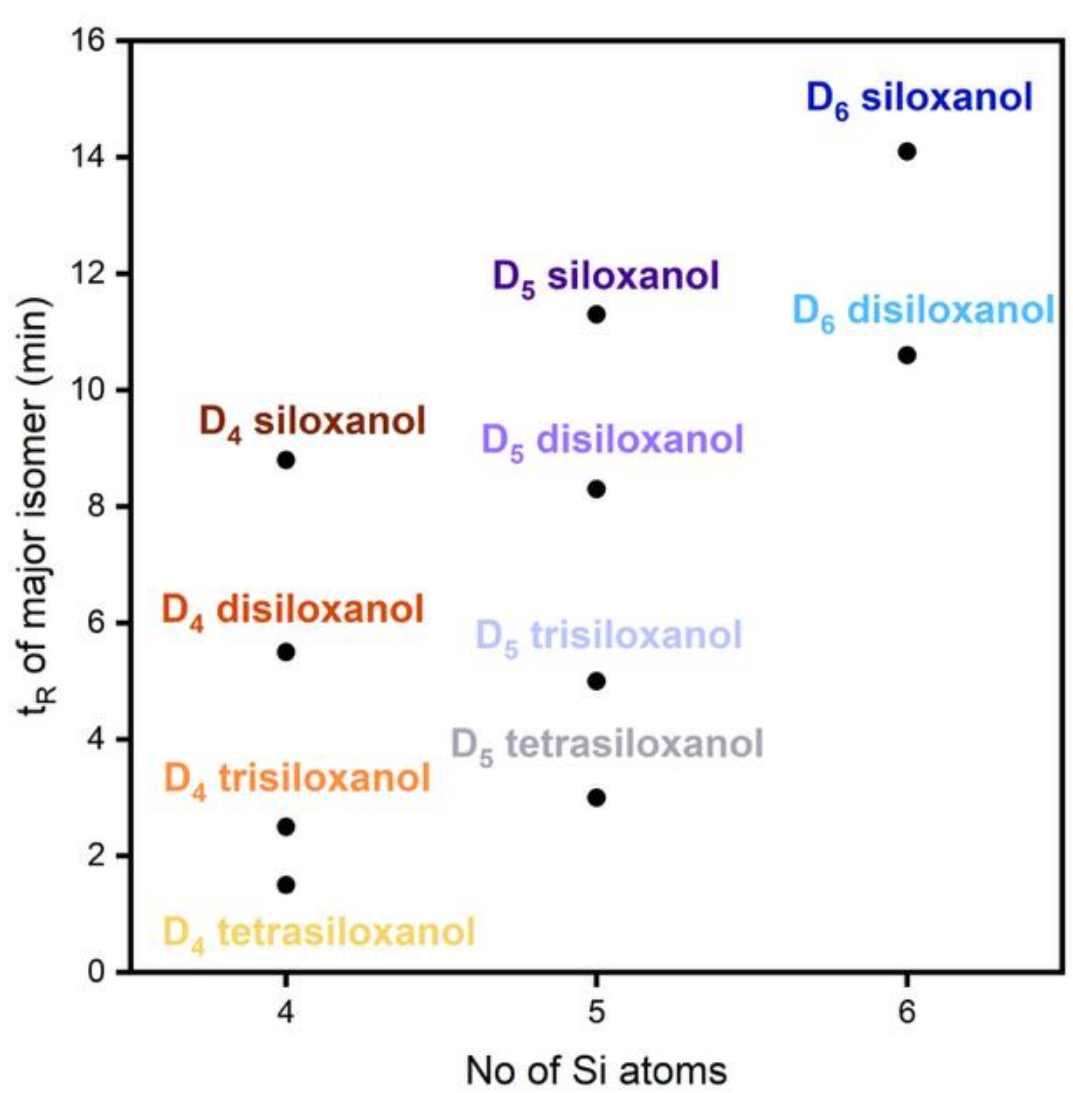

Figure S2: Retention times of the major oVMS signals listed in Table 1 (marked with stars in Figure S1) plotted against the number of silicon atoms in their molecular formulas.

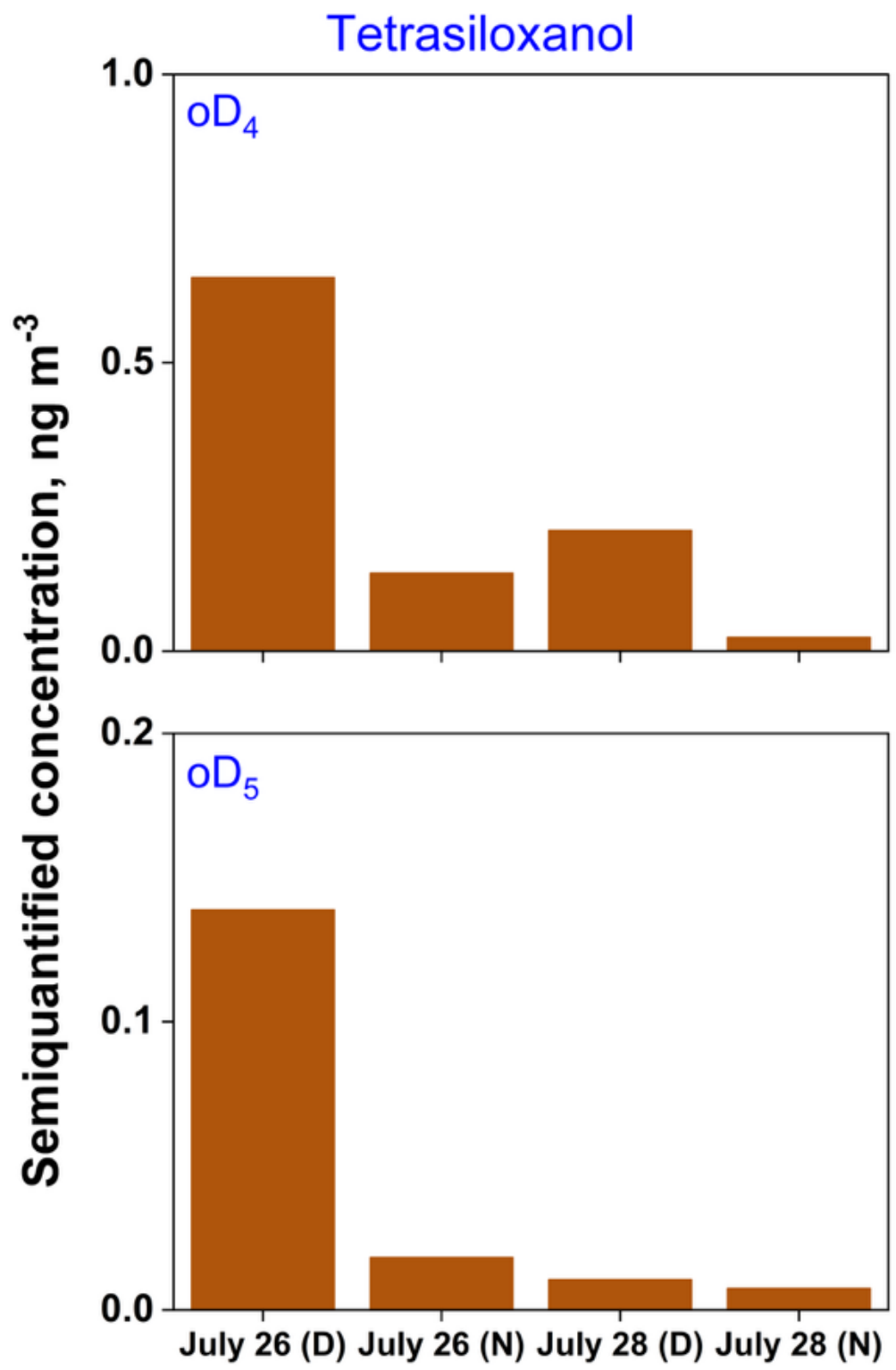

Figure S3: Semi-quantified concentrations (ng m^{-3}) of tetrasiloxanol of oD_4 and oD_5 in NYC on July 26 and 28, 2022 in the particle phase (QFF_f).

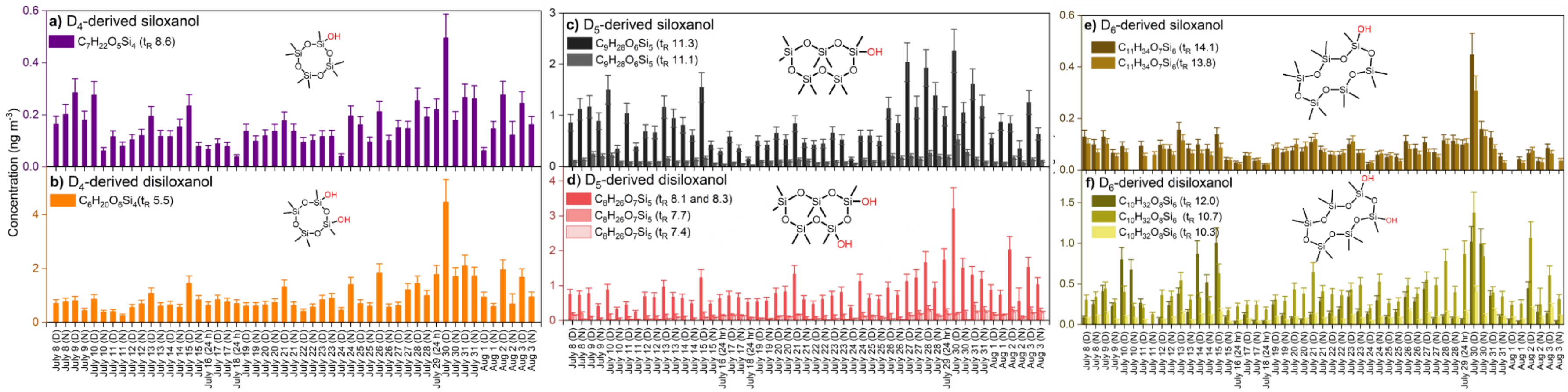

Figure S4: Time series of D_4, D_5, and D_6 oVMS in NYC, semi-quantified against tris(t-butoxy)silanol with proposed structures based on MS/MS. Panels (a, c, e) show concentrations of siloxanols derived from D_4, D_5, and D_6, respectively. Panels (b, d, f) present the corresponding disiloxanol concentrations.

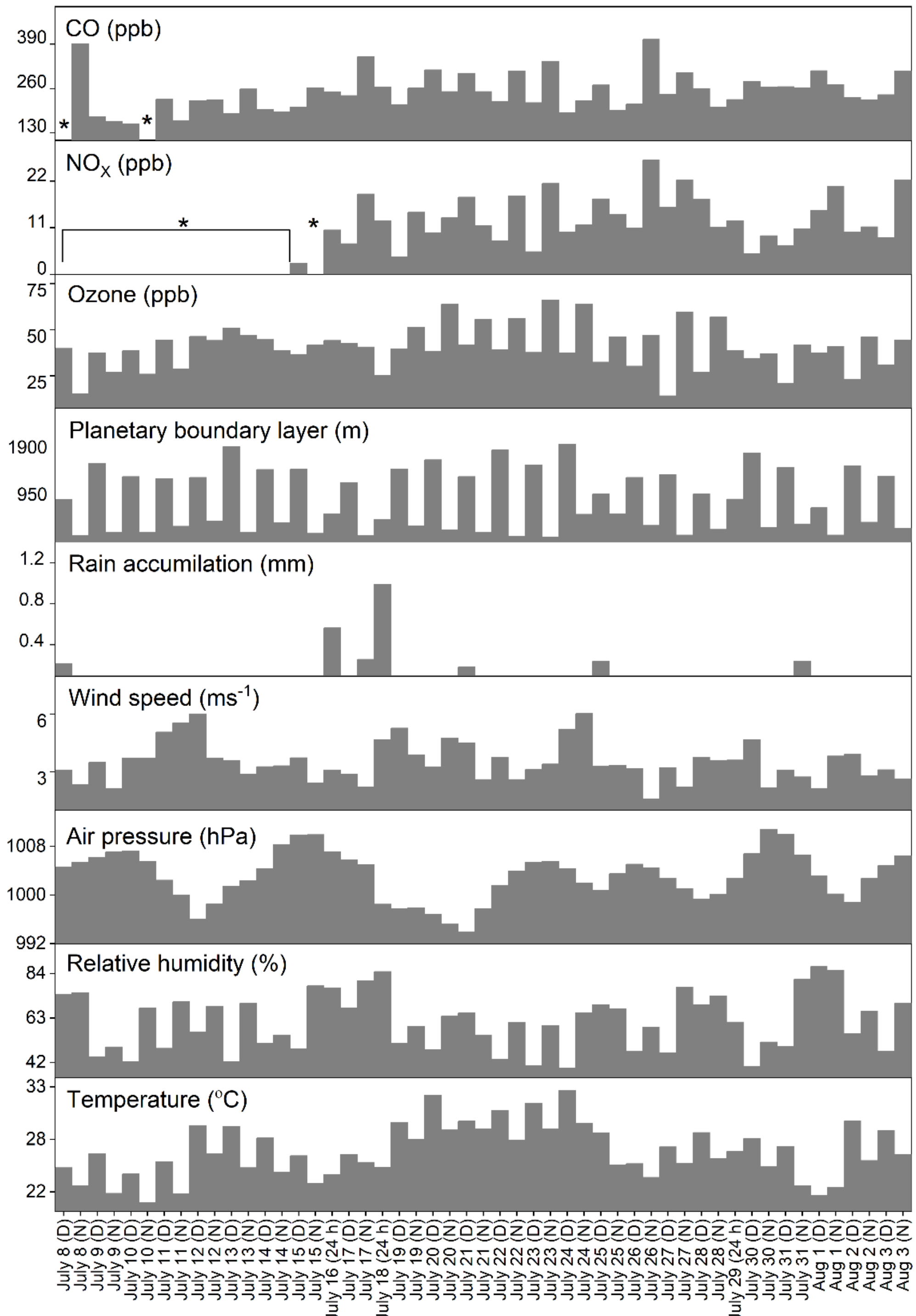

Figure S5: Average meteorological and trace gas measurements collected during the 2022 NYC-METS campaign. Periods with unavailable data are indicated by black stars.

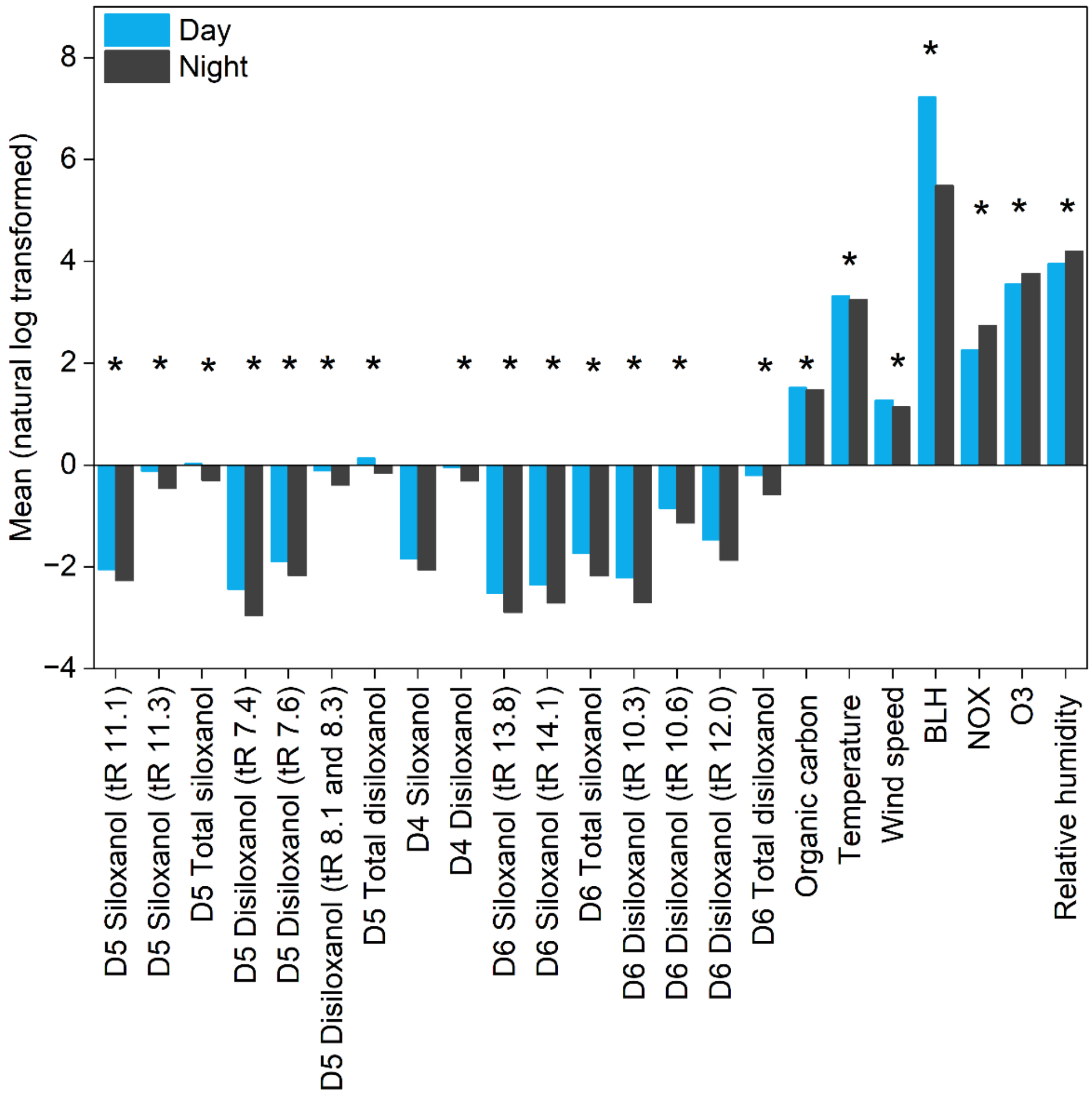

Figure S6: Comparison of natural log-transformed mean concentrations between day and night samples. Black stars indicate statistically significant differences between day and night ($p < 0.05$), determined by paired *t*-tests for normally distributed data or Wilcoxon signed-rank test for non-normal data.

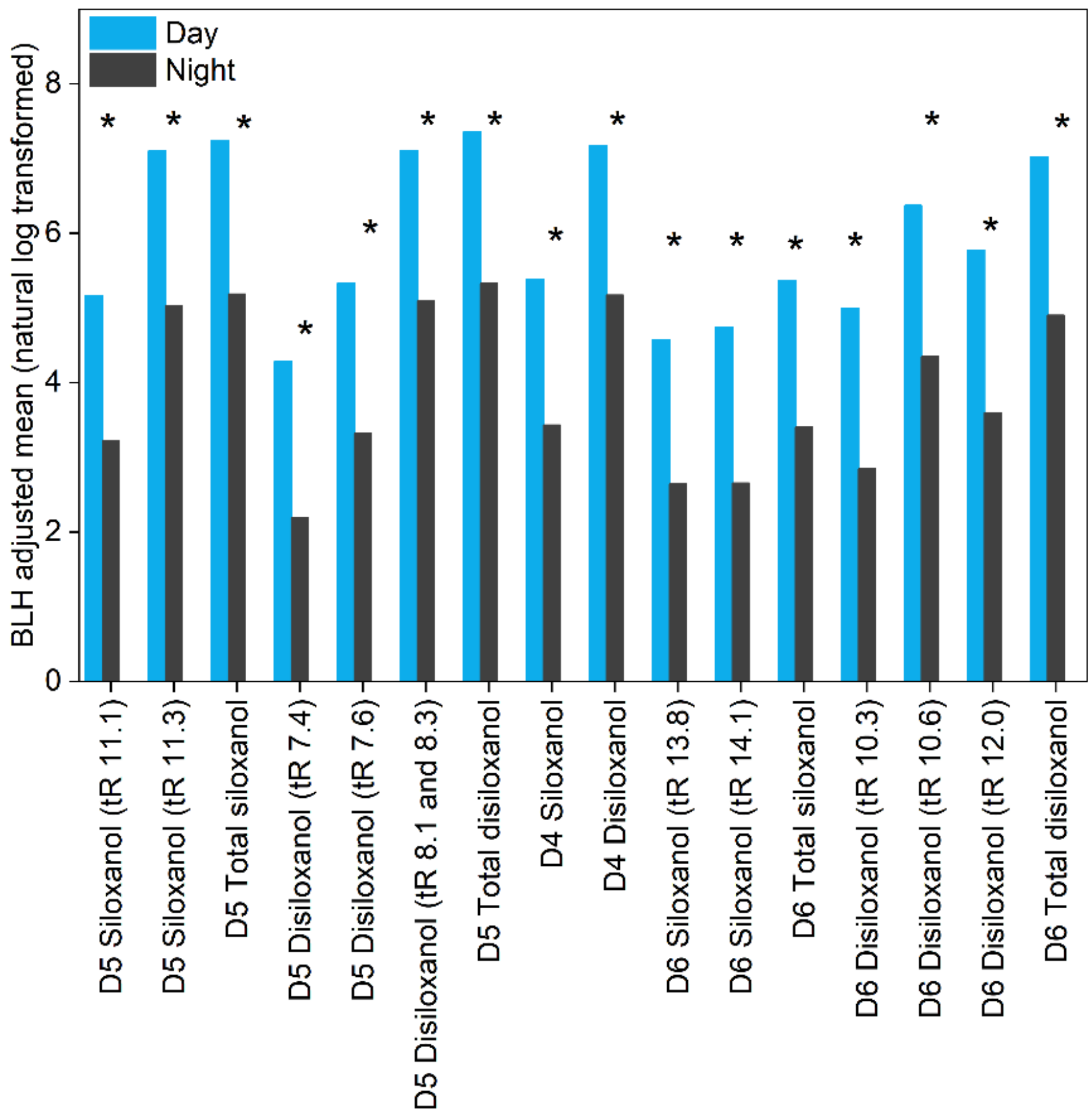

Figure S7: Comparison of natural log-transformed mean concentrations between day and night samples corrected for BLH by multiplying individual concentration by BLH. Black stars indicate statistically significant differences between day and night ($p < 0.05$), determined by paired *t*-tests for normally distributed data or Wilcoxon signed-rank test for non-normal data.

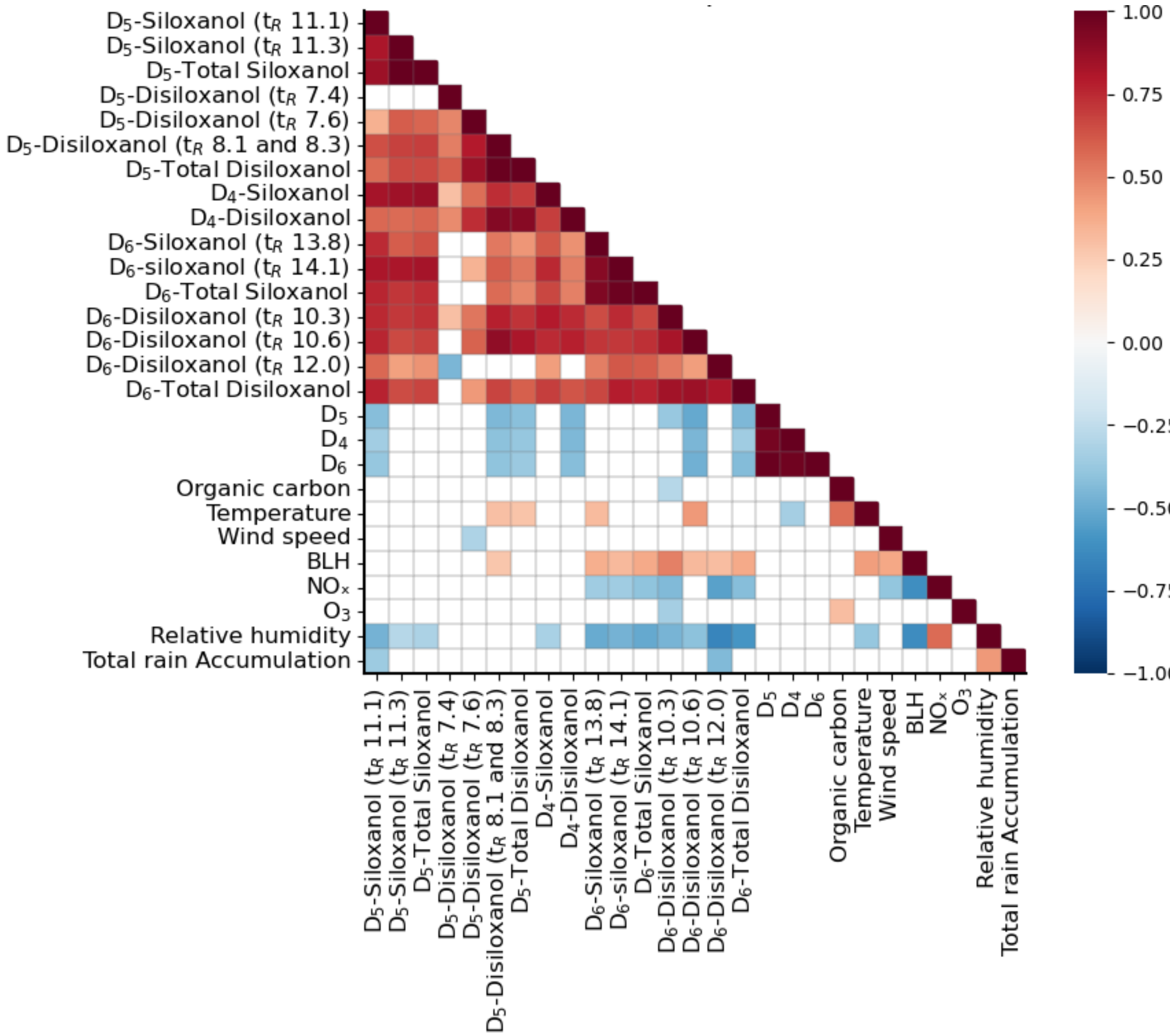

Figure S8: Pearson Correlation plot. Correlations with *p*-values greater than 0.05 were considered not significant at the 95% confidence interval and are left blank in the correlation plot.

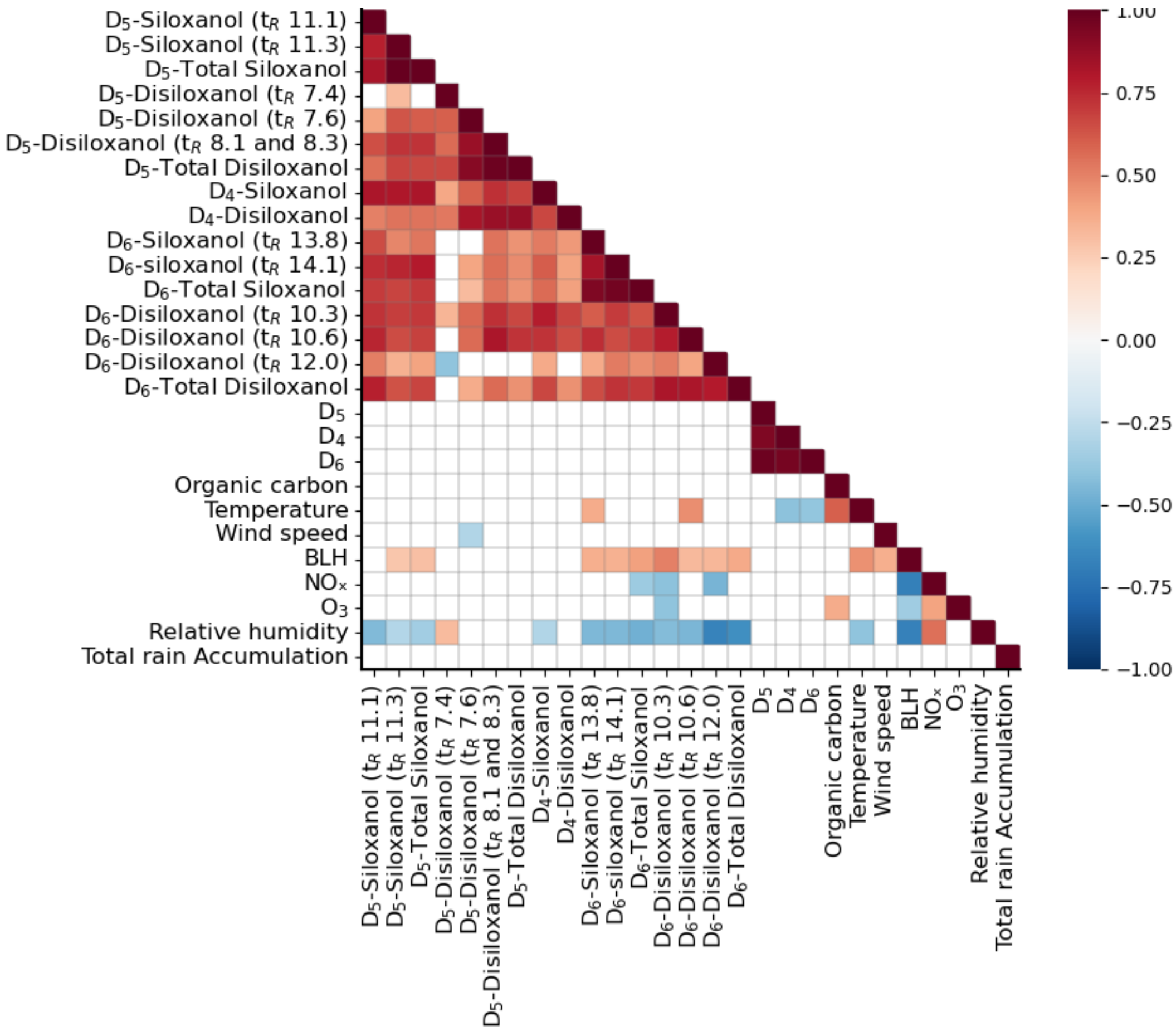

Figure S9: Spearman correlation plot. Correlations with *p*-values greater than 0.05 were considered not significant at the 95% confidence interval and are left blank in the correlation plot.

Table S1: Relative ratios of oVMS by precursor (oD_4, oD_5, oD_6) and comparison to VMS homolog ratios in NYC

	Ratio
D_5/D_4	3.3
oD_5/oD_4	2.7
D_6/D_5	0.05
oD_6/oD_5	0.2

Table S2: Day-night significance tests (paired *t*-test or Wilcoxon signed-rank test) on log-transformed variables. Bold $p < 0.05$ indicate significant differences.

Variable	Day vs night significance test	Day vs night *p* value [natural log data]
D_5 Siloxanol (t_R 11.1), ng m^{-3}	Paired-*t*-test	**0.02**
D_5 Siloxanol (t_R 11.3), ng m^{-3}	Paired-*t*-test	**0.01**
D_5 Total siloxanol, ng m^{-3}	Paired-*t*-test	**0.01**
D_5 Disiloxanol (t_R 7.4), ng m^{-3}	Paired-*t*-test	**0.01**
D_5 Disiloxanol (t_R 7.6), ng m^{-3}	Paired-*t*-test	**0.04**
D_5 Disiloxanol (t_R 8.1 and 8.3), ng m^{-3}	Paired-*t*-test	**0.01**
D_5 Total disiloxanol, ng m^{-3}	Paired-*t*-test	**0.01**
D_4 Siloxanol, ng m^{-3}	Paired-*t*-test	0.11
D_4 Disiloxanol, ng m^{-3}	Paired-*t*-test	**0.02**
D_6 Siloxanol (t_R 13.8), ng m^{-3}	Wilcoxon signed-rank test	**5.04E-4**
D_6 Siloxanol (t_R 14.1), ng m^{-3}	Wilcoxon signed-rank test	**5.85E-4**
D_6 Total siloxanol, ng m^{-3}	Wilcoxon signed-rank test	**1.46E-4**
D_6 Disiloxanol (t_R 10.3), ng m^{-3}	Paired-*t*-test	**3.25E-4**
D_6 Disiloxanol (t_R 10.6), ng m^{-3}	Paired-*t*-test	**0.01**
D_6 Disiloxanol (t_R 12.0), ng m^{-3}	Paired-*t*-test	0.05
D_6 Total disiloxanol, ng m^{-3}	Paired-*t*-test	**3.84E-3**
Organic carbon (µg m^{-3})	Paired-*t*-test	0.46
Temperature (°C)	Paired-*t*-test	**4.38E-8**
Wind speed (m s^{-1})	Paired-*t*-test	**0.04**
Planetary boundary layer height (m)	Paired-*t*-test	**3.54E-14**
NO_X (ppb)	Paired-*t*-test	**4.86E-3**
O_3 (ppb)	Wilcoxon signed-rank test	**0.03**
Relative humidity (%)	Wilcoxon signed-rank test	**1.31E-5**

Table S3: Day-night significance tests (paired *t*-test or Wilcoxon signed-rank test) on adjusted log-transformed variables. Bold $p < 0.05$ indicate significant differences.

Variable	Day vs night significance test	Day vs night *p* value [BLH adjusted natural log data]
D_5 Siloxanol (t_R 11.1), ng m^{-3}	Paired-*t*-test	**6.35E-12**
D_5 Siloxanol (t_R 11.3), ng m^{-3}	Paired-*t*-test	**1.59E-12**
D_5 Total siloxanol, ng m^{-3}	Paired-*t*-test	**1.06E-12**
D_5 Disiloxanol (t_R 7.4), ng m^{-3}	Wilcoxon signed-rank test	**4.01E-5**
D_5 Disiloxanol (t_R 7.6), ng m^{-3}	Paired-*t*-test	**1.38E-12**
D_5 Disiloxanol (t_R 8.1 and 8.3), ng m^{-3}	Paired-*t*-test	**1.63E-12**
D_5 Total disiloxanol, ng m^{-3}	Paired-*t*-test	**7.74E-12**
D_4 Siloxanol, ng m^{-3}	Paired-*t*-test	**8.72E-12**
D_4 Disiloxanol, ng m^{-3}	Paired-*t*-test	**1.42E-12**
D_6 Siloxanol (t_R 13.8), ng m^{-3}	Wilcoxon signed-rank test	**5.96E-7**
D_6 Siloxanol (t_R 14.1), ng m^{-3}	Wilcoxon signed-rank test	**8.34E-7**
D_6 Total siloxanol, ng m^{-3}	Wilcoxon signed-rank test	**5.96E-7**
D_6 Disiloxanol (t_R 10.3), ng m^{-3}	Paired-*t*-test	**5.52E-12**
D_6 Disiloxanol (t_R 10.6), ng m^{-3}	Paired-*t*-test	**2.29E-12**
D_6 Disiloxanol (t_R 12.0), ng m^{-3}	Paired-*t*-test	**1.24E-8**
D_6 Total disiloxanol, ng m^{-3}	Paired-*t*-test	**9.82E-12**

Table S4: Anderson–Darling normality results on log-transformed variables. Bold Anderson-Darling statistics indicate variables that are normally distributed.

Variable	N (Sample size)	Anderson-Darling statistic	Critical value (at $\alpha = 0.05$)
D_5-Siloxanol (t_R 11.1), ng m^{-3}	51	**0.36**	0.74
D_5-Siloxanol (t_R 11.3), ng m^{-3}	51	**0.26**	0.74
D_5-Total siloxanol, ng m^{-3}	51	**0.23**	0.74
D_5-Disiloxanol (t_R 7.4), ng m^{-3}	46	0.83	0.74
D_5-Disiloxanol (t_R 7.6), ng m^{-3}	51	**0.43**	0.74
D_5-Disiloxanol (t_R 8.1 and 8.3), ng m^{-3}	51	**0.36**	0.74
D_5-Total disiloxanol, ng m^{-3}	51	**0.29**	0.74
D_4-Siloxanol, ng m^{-3}	51	**0.22**	0.74
D_4-Disiloxanol, ng m^{-3}	51	1.01	0.74
D_6-Siloxanol (t_R 13.8), ng m^{-3}	49	0.78	0.74
D_6-Siloxanol (t_R 14.1), ng m^{-3}	47	**0.61**	0.74
D_6-Total siloxanol, ng m^{-3}	49	0.96	0.74
D_6-Disiloxanol (t_R 10.3), ng m^{-3}	50	**0.30**	0.74
D_6-Disiloxanol (t_R 10.6), ng m^{-3}	51	**0.36**	0.74
D_6-Disiloxanol (t_R 12.0), ng m^{-3}	50	**0.29**	0.74
D_6-Total disiloxanol, ng m^{-3}	51	**0.30**	0.74
D_5 (ng m^{-3})	34	**0.57**	0.72
D_4 (ng m^{-3})	34	**0.47**	0.72
D_6 (ng m^{-3})	34	**0.39**	0.72
Organic carbon (ug m^{-3})	51	**0.28**	0.74
Temperature (°C)	51	**0.32**	0.74
Wind speed (m s^{-1})	50	**0.5**	0.74
Boundary layer height (m)	51	1.71	0.74
NO_X (ppb)	36	**0.48**	0.72
O_3 (ppb)	51	1.20	0.74
Relative humidity (%)	51	**0.61**	0.74
Total rain accumulation (mm)	35	1.42	0.72

Table S5: Average estimates of $PM_{2.5}$ SOA, SOC, and percent contribution of D_4, D_5, and D_6-derived SOC to OC (± one standard deviation) in NYC. Results are shown for July 26 and July 28 (samples with backup filters) with paired and average artifact corrections.

	**July 26 and July 28, 2022 Average (with paired artifact correction, n=4)**			**July 26 and July 28, 2022 Average (with average artifact correction, n=4)**		
**Precursor**	oVMS SOA (ng m^{-3})	oVMS SOC (ngC m^{-3})	oVMS OC (%)	oVMS SOA (ng m^{-3})	oVMS SOC (ngC m^{-3})	oVMS OC (%)
**D_4**	5.1 (2.4)	1.2 (0.7)	0.04 (0.03)	5.1 (3.2)	1.2 (0.8)	0.04 (0.03)
**D_5**	5.5 (1.7)	1.4 (0.4)	0.04 (0.02)	5.6 (1.8)	1.4 (0.5)	0.04 (0.01)
**D_6**	3.1 (1.0)	0.8 (0.3)	0.02 (0.01)	3.0 (0.8)	0.8 (0.2)	0.02 (0.01)
**Sum**	13.7 (4.5)	3.5 (1.1)	0.10 (0.05)	13.6 (4.9)	3.5 (1.2)	0.10 (0.05)

Table S6: Comparison of average estimates of $PM_{2.5}$ SOA, SOC, and percent contribution of oVMS-derived SOC to OC (± one standard deviation) in NYC using chamber and OFR-derived tracer fractions. Results are shown for July 26 and July 28 (samples with backup filters).

	f_{oD5}	oVMS SOA (ng m^{-3})	oVMS SOC (ngC m^{-3})	oVMS OC (%)
Chamber (<1 day of atmospheric aging)	1.01	15.4±5.3	3.9±1.3	0.12±0.05
OFR (4 days of atmospheric aging)	2.09	7.4±2.6	1.9±0.6	0.06±0.03

References

(1) Lewine, H. R.; Meepage, J. N.; Welker, J. K.; Stanier, C. O.; Stone, E. A.; Browne, E. C. Secondary organic aerosol formation from early-generation oxidation products of decamethylcyclopentasiloxane depends on seed aerosol composition. *Environ. Sci. Atmos.* **2025**, *5* (11). DOI: 10.1039/D5EA00063G.

(2) Mohammadi, S.; Meepage, J. N.; Brunet, C. E.; Stone, E. A.; Marek, R. F.; Hornbuckle, K. C.; Browne, E. C.; Stanier, C. O. Decamethylcyclopentasiloxane (D5) oxidation: product chemistry, influence of RO2 fate, and secondary aerosol production *ACS ES&T Air* **2026**. DOI: 10.1021/acsestair.6c00037.

(3) Wu, Y.; Johnston, M. V. Aerosol Formation from OH Oxidation of the Volatile Cyclic Methyl Siloxane (cVMS) Decamethylcyclopentasiloxane. *Environ. Sci. Technol.* **2017**, *51* (8), 4445-4451. DOI: 10.1021/acs.est.7b00655.

(4) Wu, Y.; Johnston, M. V. Molecular Characterization of Secondary Aerosol from Oxidation of Cyclic Methylsiloxanes. *J. Am. Soc. Mass Spectrom.* **2016**, *27* (3), 402-409. DOI: 10.1007/s13361-015-1300-1.

(5) EPA. *Air Quality System Data Mart.* https://www.epa.gov/outdoor-air-quality-data.
